# Light on Catalytic Reaction Mechanisms: Uncovering the Conformation of Thiourea-Based Organocatalysts and Their Interaction with Nitroolefins Using Mid-infrared Spectroscopy

**DOI:** 10.1021/acs.jpclett.5c01093

**Published:** 2025-06-11

**Authors:** Piero Ferrari, Alexander K. Lemmens, Wybren Jan Buma

**Affiliations:** + HFML-FELIX, 6029 Radboud University, Nijmegen 6525 ED, The Netherlands; ‡ Molecular Photonics, Van ’t Hoff Institute for Molecular Sciences, Faculty of Science, 1234 University of Amsterdam, 1090 GD Amsterdam, The Netherlands

## Abstract

Thiourea-based organocatalysts offer a wide variety of benefits over conventional metal-based catalysts; among other things, they are cheap, harmless, and highly enantioselective. At the core of their catalytic activity is the ability to simultaneously coordinate reactants through hydrogen bonds and lower energy barriers. Detailed insight into the structure and conformational heterogeneity of the catalyst together with the coordination of the reactants is thus key to understanding the mechanisms by which organocatalysts perform their function. Here, using the Takemoto organocatalyst and 1-(2-nitroethyl)­naphthalene as a prototypical catalyst–reactant system involved in Michael addition reactions, we show that mid-infrared spectroscopy covering the wide spectral range from 650 to 3500 cm^–1^ combined with molecular beam experiments and density functional theory calculations is a powerful approach to meet these challenges. We determine the precise geometry adopted by the catalyst–nitroolefin complex, thereby providing direct information about the hydrogen bonds involved. Moreover, we solve a long-standing problem of the preferred conformation of the bare catalyst by providing an unambiguous determination of its structure. The general applicability of our approach holds great promise for extending it to elucidate other relevant complex reactions involving (organo)­catalysts.

## Graphical abstract

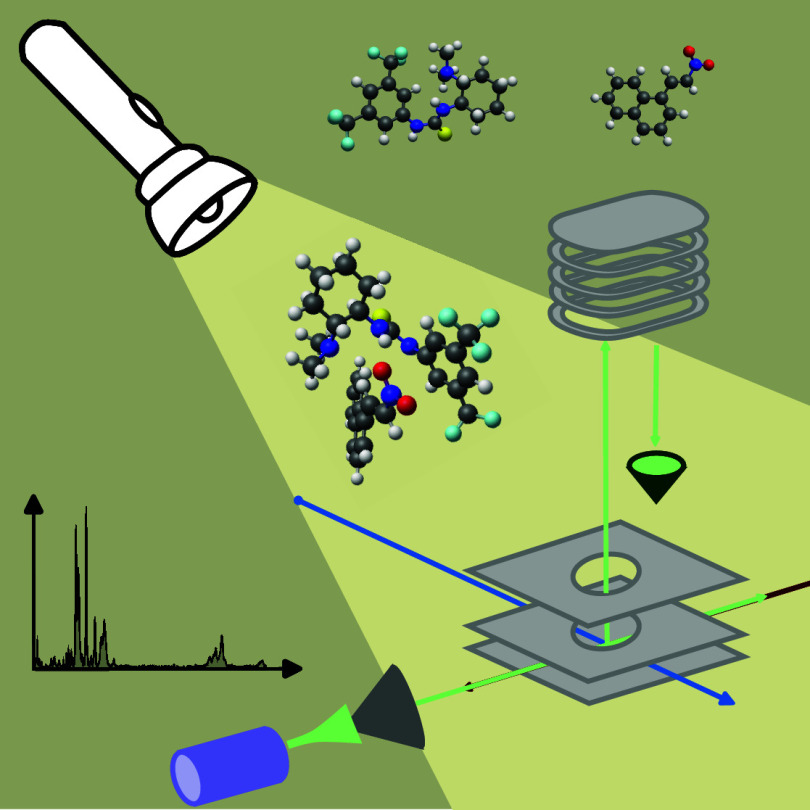

Catalysts have shaped our society, with applications in fields as diverse as food production, pharmaceutical synthesis, and sustainable energy. Commonly, catalysts are metal-based, making them expensive, environmentally unfriendly, and often toxic. In recent years, however, organocatalysts have gained a great deal of attention, given their potential to be cheap, stable, harmless, and environmentally friendly,^1^ as well as providing high enantioselectivity.^2^ To date, many organocatalysts have been shown to be effective catalysts in, for example, the Diels–Alder reaction,^3^ the Mannich reaction,^4^ and the Michael addition.^5^ An important class of organocatalysts are those operating via hydrogen bonding, where the catalytic center is an asymmetrically substituted urea or thiourea motif.^6–8^ Inspired by the functionality of enzymes in biological systems, such organocatalysts rely on multiple hydrogen bonds to coordinate precursors, as well as to stabilize transition states found along the reaction pathways, thus lowering the energy barriers.^9^

A famous example of a thiourea-based organocatalyst is Takemoto’s catalyst,^10^ which has been shown to catalyze the important enantioselective Michael addition of malonates to nitroolefins.^11^ This catalyst is a so-called “bifunctional” catalyst, with its activity relying on the formation of hydrogen bonds with both reactants simultaneously.^12^ This offers wider control over coordination than cases with a single hydrogen bond, thus allowing the stereoselectivity to be steered.^13^ At such, the functionality of the catalyst strongly relies on its conformation as well as on the binding coordination of the reactants.

In the catalyst, a variety of conformations are possible, which are grouped into distinct families based on the orientation of the N–H and C=S bonds of the central thiourea.^14^ Experiments either on crystals or in solution have been used to address the preferred conformation of the catalyst,^15,16^ often leading to conflicting results, attributed to the flexible and dynamical geometry of the catalyst, which has even been shown to depend on the solvent involved in the reaction.^17^ Moreover, from a computational point of view, density functional theory (DFT) calculations have been used to predict the lowest-energy conformation of the catalyst.^18,19^ In this case, however, calculations rely strongly on the combination of functionals and basis sets employed, making an assignment of the preferred conformation of the catalyst unreliable. Nevertheless, the consensus is that the activity of the catalyst is uniquely dependent on its conformation. Similarly, experiments have been combined with DFT calculations to address the coordination of reactants onto the catalyst, but with similar bottlenecks to obtain precise and reliable information about the structure and the relevant inter- and intramolecular interactions at play.^20^ Such information is, however, crucial for the rational design of new and more efficient organocatalysts.

Infrared spectroscopy studies under the pristine conditions provided by molecular beams are preeminently suited to obtain such information,^21^ as they provide detailed fingerprints of structure,^22^ intra- and intermolecular interactions,^23^ and hydrogen bond formation.^24^ Gas-phase infrared spectroscopy has indeed been employed in the past to determine the molecular structure of both metal-based catalysts^25^ and organocatalysts.^26,27^ Important to note is that these studies focused on ionic species. Similar studies on neutral catalysts are significantly more difficult and have not yet been reported.

Here, we study Takemoto’s catalyst in a molecular beam of neutrals, as well as its complex with 1-(2-nitroethyl)­naphthalene, a nitroolefin shown to have a high reaction yield in its Michael addition to malonates.^10^ Infrared (IR) spectroscopy experiments are performed by merging the molecular beam with the laser light of the free electron laser FELIX (Nijmegen, The Netherlands), which gives access to a wide spectral range, from 650 to 3500 cm^–1^. This is crucial, since key fingerprint vibrational features of the catalyst are found in this range, as well as vibrational modes that are sensitive to the subtle interactions with the nitroolefin. We will show that this approach enables us to characterize the catalyst and the catalyst–reactant system in unprecedented detail and elucidate the mode of action of the catalyst. As such, this study breaks new ground for similar studies on other organocatalysts as well as metal-based catalysts and their complex catalytic reactions.

## Methods

*Experimental Methods*. Experiments were performed in the gas phase employing a neutral molecular beam, formed via the technique of laser desorption.^28^ In short, a powder of Takemoto’s catalyst, 1-[3,5-bis­(trifluoromethyl)­phenyl]-3-[(1*R*,2*R*)-(−)-2-(dimethylamino)­cyclohexyl]­thiourea (Cat), alone or a combination of the catalyst with 1-(2-nitroethyl)­naphthalene (Nitro) is mixed in a 1:1 ratio with black carbon and pressed onto the surface of a movable graphite bar. The catalyst powder was purchased from Sigma-Aldrich, whereas the 1-(2-nitroethyl)­naphthalene was synthesized in house (see the Supporting Information). The graphite bar is placed at the exit of a pulsed supersonic valve that injects a pulse of Ar gas at a backing pressure of 4 bar. Shortly after the Ar pulse, the pressed molecules are desorbed from the graphite bar using the fundamental light of a Nd:YAG laser (1064 nm, 1 mJ/pp), mildly focused to about 1 mm, and merged with the expanding Ar gas, forming a molecular beam of neutrals. We remark that the formation of the Cat···Nitro complex depends to a major extent on how the molecular beam is generated, in particular, necessitating a relatively heavy load of Ar gas and somewhat low laser ablation energies.

Subsequently, the beam is collimated by a 1 mm skimmer, after which it enters the ionization region of a perpendicularly placed reflectron time-of-flight mass spectrometer (*m*/Δ*m* = 2200 at 400 amu), where the species present in the beam are characterized. In order to record a mass spectrum, molecules are ionized at the entrance of the mass spectrometer by using 118 nm (10.5 eV) photons generated in a Xe/Ar cell (20 mbar of Xe in 150 mbar of Ar) pumped by the tightly focused third harmonic of a Nd:YAG laser (355 nm, 20 mJ/pp). The use of the Xe/Ar cell allows for the simultaneous off-resonance ionization of the different species in the molecular beam, which means that the measured infrared spectra are recorded under the same experimental conditions. A sketch of the experimental setup is presented in the Supporting Information.

The molecular beam is then merged with the counterpropagating light of the free electron laser FELIX (Nijmegen, The Netherlands), which is timed 300 μs before ionization takes place, thus ensuring that the probed molecules are neutrals. Infrared absorption spectra are measured using infrared multiple photon dissociation (IRMPD) spectroscopy.^29^ Upon resonant excitation with a vibrational mode, multiple photon absorptions occur with the energy of each photon being promptly redistributed among all vibrational degrees of freedom of the molecule (vibrational energy redistribution (IVR)). If by this process the dissociation threshold is overcome, fragmentation is triggered, and a depletion of the signal at the mass of the molecular ion is observed. The IRMPD scheme is particularly efficient with FELIX, given the unique time structure of a free electron laser, where each macropulse (∼10 μs) consists of hundreds of micropulses (∼5 ps), which permits the absorption of many resonant photons.^30^ In order to account for signal fluctuations, the molecular beam experiment is run at 10 Hz, whereas FELIX is triggered at 5 Hz, thus allowing the alternating recording of mass spectra with and without FELIX interaction. Each mass spectrum is the average over 80 individual shots at a fixed wavenumber, while FELIX is tuned over the wide range from 650 to 3600 cm^–1^ in steps of 2 cm^–1^. The infrared intensity is defined as *I*_IR_ = −ln­(*I*/*I*_0_)/*P*, where *I* and *I*_0_ are the intensity in mass spectra with and without FELIX interaction, respectively, and *P* is the laser power. The line width of FELIX is dependent on wavelength, being a percentage (∼0.3%) of the central wavelength. This is reflected in an increasing width of the measured IR features with frequency.

*Computational Methods*. The measured infrared spectra are complemented with DFT calculations, performed with the ORCA 6.01 software package.^31^ For the bare catalyst, conformations are considered based on previous studies.^16^ Instead, for the Cat···Nitro complex, a conformational search was performed using the Global Optimizer Algorithm (GOAT) implemented in ORCA,^32^ yielding 300 conformers. Of this set, the 50 lower-energy conformers were reoptimized at the DFT level. Two functionals were employed in these calculations, PBE0 and M06-2X. Previous DFT studies have shown that M06-2X is well suited to predict the vibrational frequencies of molecules involving C–F stretches, as is the case for the Takemoto catalyst, but its performance on complexes is less good.^33^ We arrive at a similar conclusion in our analysis, as will be discussed below. We have therefore chosen to use M06-2X for calculations on the bare catalyst, while PBE0 was used for the Cat···Nitro complex. In both cases, DB3J dispersion corrections have been included, and the def2-TZVP basis set was employed. The calculations were run using the “very tight” convergency criterion for the self-consistent field (SCF) cycles and geometry optimization, as implemented in ORCA. Moreover, a very fine grid size was selected (defgrid3 option). Analytic harmonic frequencies were computed for all conformers, confirming that they are associated with a potential energy minimum. Given the frequency-dependent line width of FELIX, the computed infrared spectra that will be presented are constructed as a convolution of Gaussians centered at each vibrational frequency, with a full width at half-maximum (fwhm) of 1% of the central frequency. The harmonic frequencies are scaled by a factor of 0.96 for frequencies below 2000 cm^–1^, whereas above 2000 cm^–1^, a factor of 0.95 is employed. Cartesian coordinates of the bare catalyst, nitroolefin, and the Cat···Nitro complex are given in the Supporting Information.

A typical mass spectrum of the species in a molecular beam using a mixture of catalyst and nitroolefin powders as a precursor is presented in Figure 1. Under the employed experimental conditions of Ar gas pressure and ablation laser energy, intense peaks corresponding to the bare nitroolefin (199 amu) and the catalyst (413 amu) are observed, as well as the dimer of the catalyst (826 amu). In addition, a low-intensity feature is visible at 612 amu that corresponds to the Cat···Nitro complex. The intensity of the latter is low but, as shown below, sufficient to successfully record an infrared spectrum. The mass spectrum also shows peaks between 270 and 400 amu. The intensity of these peaks is sensitive to the power of the ablation laser. They are therefore attributed to fragments from the catalyst. In our IR absorption experiments, the power of the ablation laser is maintained as low as possible to minimize the appearance of these peaks.

**Figure 1:**
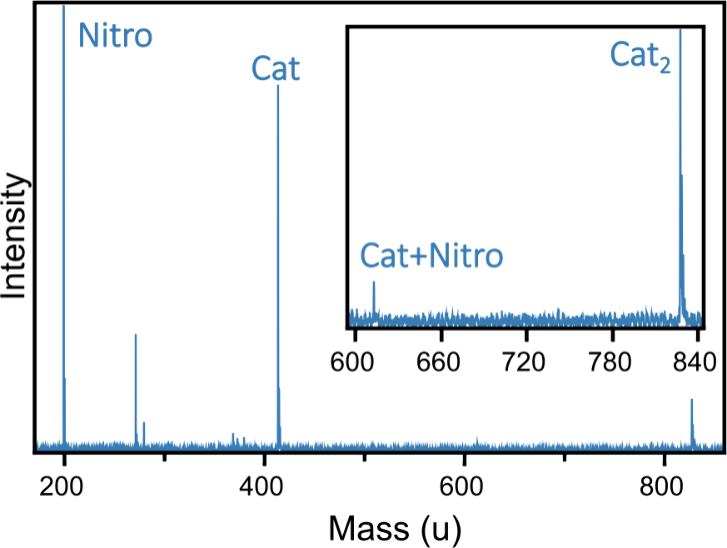
Typical mass spectrum of a molecular beam in which a mixture of catalyst and nitroolefin powders is used as a precursor. The peaks corresponding to the nitroolefin and catalyst molecules are labeled, as well as the dimer between two catalyst molecules and the Cat···Nitro complex. A close-up of the region of larger masses is shown as an inset.

First, we focus on the bare catalyst, with Figure 2a presenting the measured infrared spectrum in the range from 650 to 3500 cm^–1^. Many strong features are resolved, in particular within the ranges of 1000–1700 and 2700–3600 cm^–1^, as well as weaker bands below 1000 cm^–1^. The IR spectrum agrees well with previous measurements of Takemoto’s catalyst in solution,^16^ although the features in Figure 2a are better resolved and provide a wider spectroscopic range than the previously covered range of 1000–1800 cm^–1^. Complementary DFT calculations of the IR spectrum of the bare catalyst are performed for the five conformers concluded to be dominant in earlier work.^16^ These conformers are depicted in Figure 3, and they are labeled according to the orientation of the N–H and C=S bonds, which can be symmetric (**s**) or antisymmetric (**a**). There are therefore in general four families of configurations: **aa**, **as**, **sa**, and **ss**. Here two **aa** conformations, two **sa** conformations, and one **as** conformation are considered. As shown in previous work, the energy ordering between the conformers of the catalyst depends on the functional employed for the calculations, making a prediction of the preferred conformation based only on computations unreliable.^16^ In our calculations, M06-2X predicts conformer **sa1** as the lowest in energy, followed by **sa2** (7.7 kJ/mol), **as1** (12.5 kJ/mol), **aa2** (15.4 kJ/mol), and **aa1** (16.4 kJ/mol). Conversely, using the PBE0 functional, **sa2** becomes the lowest-energy conformer, followed by **sa1** (1.0 kJ/mol), **as1** (5.8 kJ/mol), **aa2** (7.8 kJ/mol), and **aa1** (7.8 kJ/mol). Similar mismatched energy orderings have been found when comparing results between the M06-2X and B3LYP functionals.^16^

**Figure 2:**
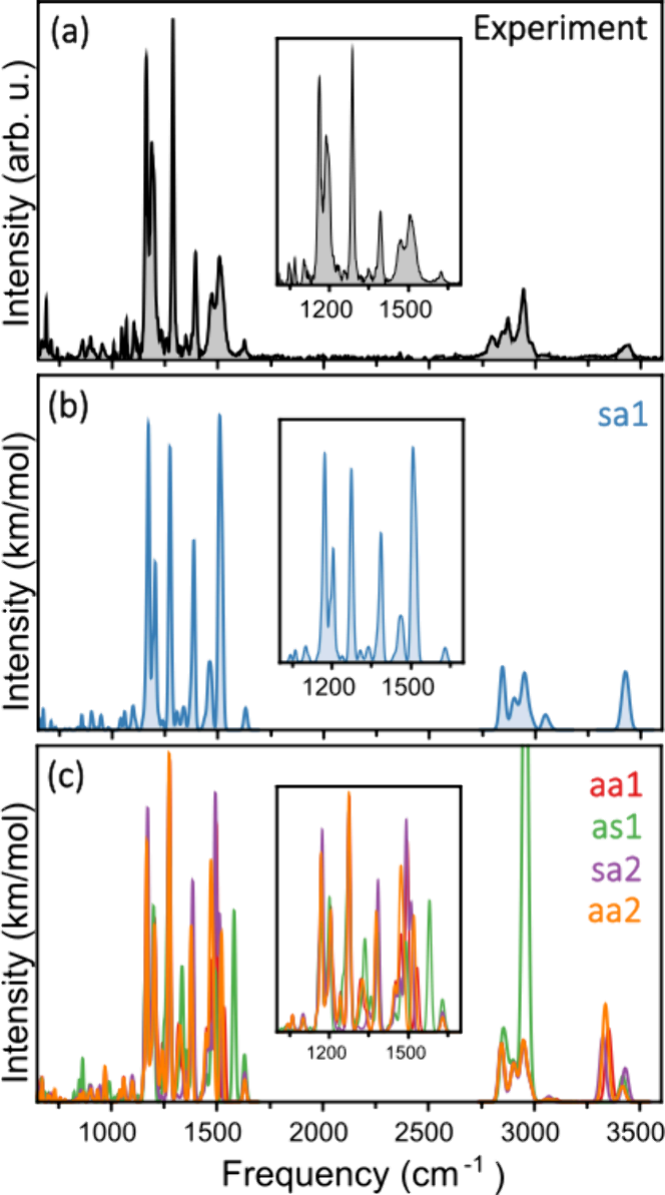
(a) Measured infrared spectrum of Takemoto’s catalyst. The increasing width of the bands with wavenumber is a consequence of the wavelength-dependent line width of FELIX. (b) Computed infrared spectrum of conformer **sa1** of the catalyst. Gaussian functions with a frequency-dependent fwhm (1% of the central frequency) are used to construct the spectrum. The M06-2X functional is employed. (c) Computed infrared spectra of conformers **aa1**, **as1**, **sa2**, and **aa2** of the catalyst. In all panels, a close-up of the region between 1000 and 1700 cm^–1^ is presented.

**Figure 3:**
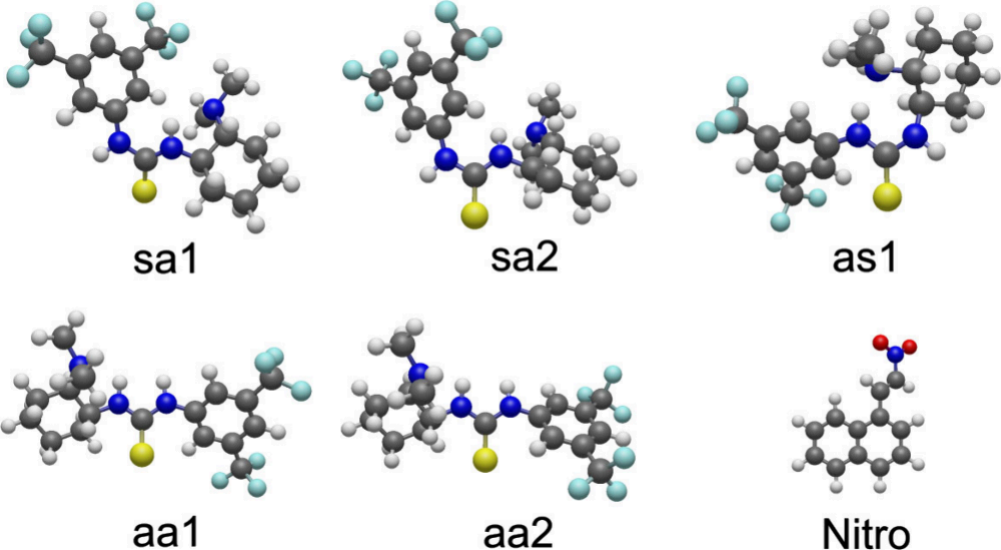
Configurations considered for the bare Takemoto catalyst. Each is labeled according to the relative orientation of the N–H and C=S bonds (**s**, symmetric; **a**, antisymmetric). In addition, the 1-(2-nitroethyl)­naphthalene structure is shown.

Gas-phase infrared spectroscopy provides a unique way to deal with this conundrum. The M06-2X-computed IR spectra of the five conformers of the catalyst are presented in panels b and c of Figure 2. and in Figure S2 in the Supporting Information. Comparison of these spectra with the experimental spectrum rapidly leads to the conclusion that the spectrum of conformer **sa1** (panel b) is in excellent agreement with the experimental spectrum. All features are reproduced, in particular the strong bands at 1161 and 1190 cm^–1^ (composed of two close modes), 1286 cm^–1^, and 1394 cm^–1^, as well as the broader bands at 1469 and 1507 cm^–1^. The last two features are predicted to each have contributions from two overlapping modes, thereby explaining their broader line widths seen in the experiment. In the range of 2600–3600 cm^–1^, the calculations also reproduce the experiment fairly well, although the relative intensities of the bands do not always match. In contrast, as one can conclude from panel c, all other conformers show one or more features that are not observed experimentally. Conformers **sa2**, **aa1**, and **aa2** predict a band around 3337 cm^–1^, but this band is absent in the experimental spectrum. Similarly, for conformer **as1**, a feature is predicted at 1581 cm^–1^ that is not present in the experiment (see the close-up) as well as a band at 2956 cm^–1^ with a much higher intensity than experimentally observed. A quantitative analysis of the agreement between the experiment and computations is based on calculations of the similarity score between the spectra. This is done via the cosine similarity score, as discussed in ref 34. This analysis yields scores 0.49, 0.43, 0.40, 0.39, and 0.33 for **sa1**, **as2**, **aa2**, **aa1**, and **as1**, respectively, thus reinforcing our assignments. We thus conclude from this analysis that under molecular beam conditions, conformer **sa1** is dominantly present.

In agreement with this conclusion are calculations of Gibbs free energies at 300 K, which allow for elucidating the possibility that multiple conformers coexist in the molecular beam. Employing the M06-2X functional and a Boltzmann factor for the weight of each conformer, **sa1** is predicted to overwhelmingly dominate the distribution with 93.8%, followed by **sa2** (3.8%), **as1** (2.1%), **aa1** (0.2%), and **aa2** (0.1%). As expected for such a distribution and shown in the Supporting Information, combining the computed IR spectra of the different conformers based on these populations yields a spectrum that essentially is the same as that of **sa1**. This information is key, as the activity of Takemoto’s catalyst is thought to be dependent on the **aa1** conformation, so most computational analyses of reaction pathways are based on **aa1**.^16,20,18^ Similarly, calculations of the stacking patterns of the catalysts itself have been performed, but focusing on **aa1**.^19^

Following, we discuss the results obtained for the Cat···Nitro complex. Despite the low concentration of this complex in the molecular beam (Figure 1), the signal was sufficient to successfully record infrared spectra. Figure 4 compares the spectra recorded for the catalyst (top), nitroolefin (bottom), and the Cat···Nitro complex (middle). Importantly, these three spectra are measured simultaneously under the same experimental conditions. Clearly, the signal-to-noise ratio in the spectrum of the complex is lower than that observed for the spectra of the bare molecules but nevertheless is more than good enough to observe well-resolved bands. Overall, the spectrum of the complex can be regarded as a mixture of the two separate species with all of the observed bands attributed either to the catalyst or to the nitroolefin. There are, however, observable shifts of peaks upon complexation. For example, the catalyst peak at 3435 cm^–1^ is red-shifted to 3382 cm^–1^ in the complex. Moreover, the two intense peaks of the nitroolefin at 1355 and 1552 cm^–1^ are slightly red-shifted to 1347 and 1545 cm^–1^, respectively. In contrast, the intense catalyst peaks between 1000 and 1700 cm^–1^ are not affected by complexation, nor are the features between 2700 and 3150 cm^–1^ of both the catalyst and the nitroolefin. In the following, we will show that these changes upon complexation allow for a unique determination of the conformational structure of the complex.

**Figure 4:**
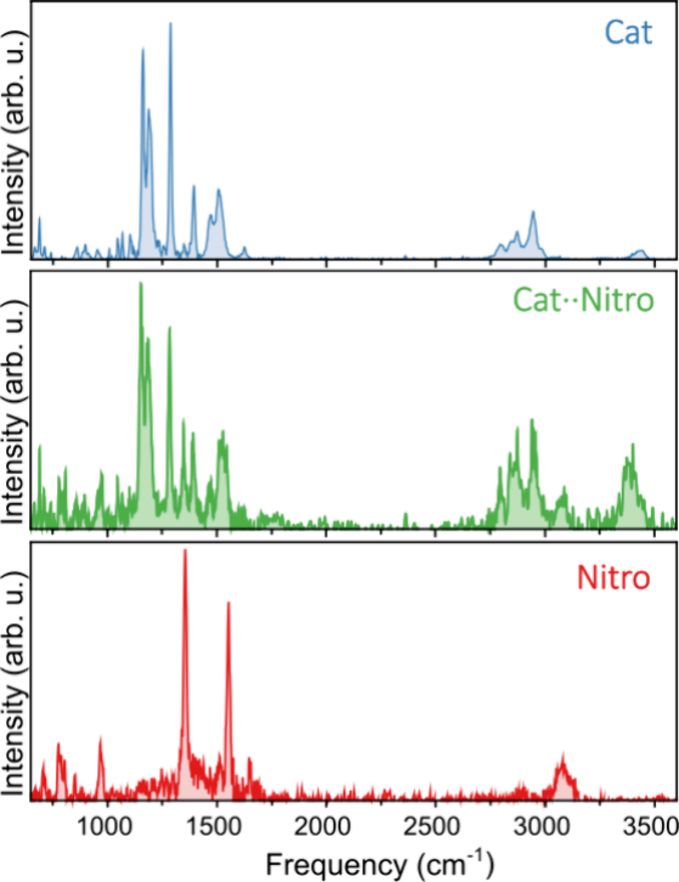
Measured infrared spectra of the bare catalyst (top) and nitroolefin (bottom) molecules. The middle panel presents the spectrum of the Cat···Nitro complex.

DFT calculations performed on the Cat···Nitro complex started with an extensive structural search of the possible conformations of the system. This search included the different coordination sites of the nitroolefin together with the possible conformations of the catalyst. As discussed in Methods, the M06-2X functional has been shown to perform well in molecules containing C–F bonds,^33^ an observation that is confirmed by our calculations of the infrared spectrum of the catalyst (Figure 2). Its performance on complexes, however, has been questioned.^16^ As discussed in detail in the Supporting Information, we furthermore have found that the M06-2X functional does not perform well in predicting the infrared spectrum of bare nitroolefin; its structure is shown in Figure 3. For this reason, various functionals were tested for calculations of the bare catalyst and nitroolefin molecules in order to find a suitable level of theory that predicts sufficiently well the infrared spectrum of both molecules. From these tests, it was concluded that in that respect PBE0 performed best. At the same time, we recall that with respect to total energies M06-2X performs better, so we rely on that functional for comparing relative energies. A list of relative energies is presented in the Supporting Information, including the M06-2X and PBE0 values.

The lowest-energy conformation of the Cat···Nitro complex (**Iso1**) is shown in Figure 5 (left), which depicts two views of its geometry. In **Iso1**, the catalyst undergoes a conformational change from **sa1** to **sa2**, which allows one of the oxygen atoms of the nitroolefin to form a hydrogen bond with one of the N–H pairs in the catalyst while leaving space for the naphthalene unit away from the two ring structures in the catalyst. Furthermore, we also consider **Iso38** (see Table S1), which has thus far been predicted as an important step in the reaction pathway of the Michael addition and therefore has been the subject of previous studies, although primarily focusing on DFT calculations.^16,18,20^ In **Iso38**, the catalyst adopts conformation **aa1** such that both O atoms in the nitroolefin can interact with the N–H pairs of the catalyst. In our computations at the M06-2X level, **Iso38** lies 46.1 kJ/mol above **Iso1**. Instead, at the PBE0 level, **Iso38** is the lowest-energy conformation, with **Iso1** lying 8.6 kJ/mol higher.

**Figure 5:**
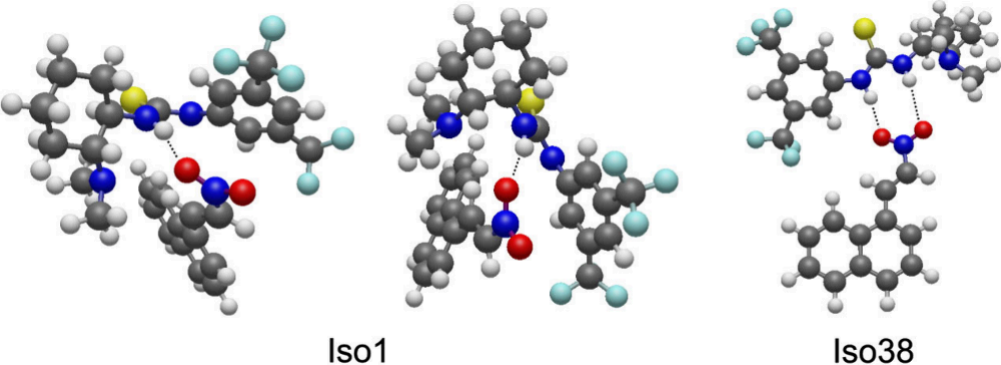
Conformations of two isomers of the Cat···Nitro complex. **Iso38** is 46.1 kJ/mol higher in energy than **Iso1**. Two views of **Iso1** are depicted. Dashed lines highlight the hydrogen bond interaction between oxygen and the N–H pairs.

The best agreement between the experimental infrared spectrum of the Cat···Nitro complex and the computations of vibrational spectra of all studied conformers of the complex is that with **Iso1**. Figure 6 presents for this purpose the comparison between the experiment and calculation in the two frequency ranges where bands are observed. In this figure, the experimental data and the computed **Iso1** IR absorption spectrum are colored black and green, respectively. Overall, the calculations reproduce quite well the experimental spectrum, although, in particular, in the range from 1100 to 1300 cm^–1^ a few peaks are slightly shifted. In the Supporting Information, we show that those shifts are not due to an incorrect prediction of the conformation of the Cat···Nitro complex but arise because the PBE0 calculations do not perfectly reproduce the band positions of the bare catalyst and nitroolefin. Figure 6 also displays the spectrum calculated for **Iso38** (red). This spectrum features very strong bands at 1356, 3330, and 3355 cm^–1^. Since such bands are not observed in the experimental spectrum, we conclude that **Iso38** is not present in the molecular beam. A similar conclusion can be drawn for other conformations of the Cat···Nitro complex, as for these conformations also features are predicted that are absent in the experimental spectrum. As discussed in the analysis of the bare catalyst, similarity scores between the experiment and computations were calculated for the different conformers of the Cat···Nitro complex. Here, too, the quantitative analysis agrees with our conclusions, with **Iso1** having the larger score (0.42) among all considered Cat···Nitro conformers. For **Iso38**, the lower score of 0.30 is found. Finally, based on relative zero-point-corrected energies at the M06-2X level of theory, the population of the different conformers was estimated. In this case, **Iso1** dominates with 95%, with **Iso38** having no contribution (0%) on the ensemble. As for the bare catalyst, the Supporting Information presents a Boltzmann-weighted infrared spectrum, showing negligible differences from the spectrum of **Iso1**.

**Figure 6:**
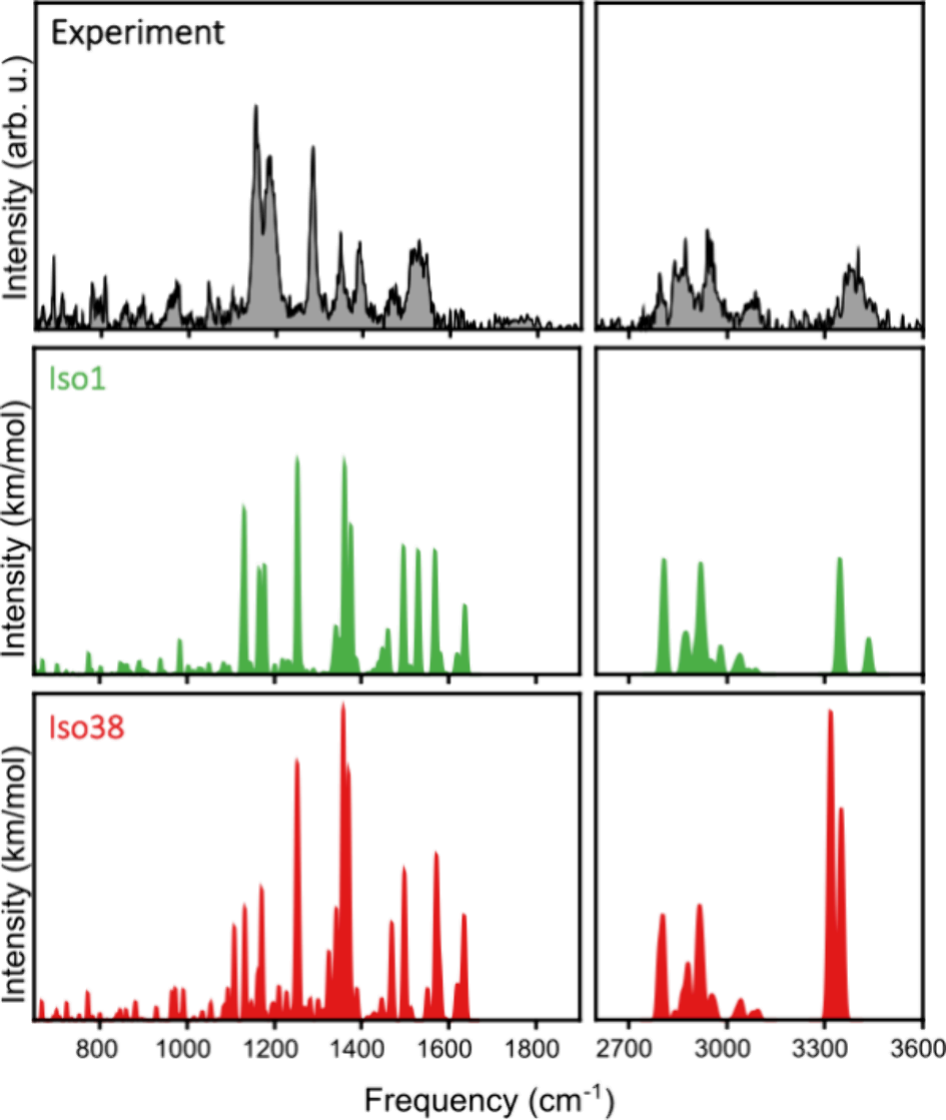
Comparison of the experimental spectrum of the Cat···Nitro complex with calculations of **Iso1** (green) and **Iso38** (red).

The observed shifts upon complexation provide information about the intermolecular interactions in the Cat···Nitro complex. The catalyst peak observed at 3435 cm^–1^ corresponds to the N–H stretches, which in the **sa1** conformation are close in frequency, but not exactly degenerate. In the complex, however, the two N–H stretches are clearly separate, as seen in the calculation of **Iso1** in the right panel of Figure 6. In **Iso1**, one of the N–H stretches is red-shifted, while the other remains unaffected. Since the resolution in the experimental spectrum is not high enough to resolve both bands, the overall effect is a red-shift of the band as observed experimentally. This suggests that in the Cat···Nitro complex only one N–H is involved in hydrogen bonding with an O atom of the nitroolefin. In **Iso38**, instead, both N–H groups are involved in hydrogen bonding, leading to a red-shift of both N–H stretches. This is in disagreement with the experimental observations. Similarly, the experiment shows red-shifts of the peaks associated with the nitroolefin, which are found at 1347 and 1545 cm^–1^ in the complex. The calculations assign these modes as the NO_2_ symmetric and asymmetric stretch modes, which are also affected by the formation of a hydrogen bond with the catalyst. Nevertheless, the effect is weaker in this case, as only one of the O atoms of the nitroolefin is expected to participate in the bonding. If both oxygens were involved in bonding, such as in **Iso38**, this would lead to a strong feature at 1358 cm^–1^, which is not observed in the experiment. Finally, we consider the modes between 2700 and 3150 cm^–1^ and the features between 1000 and 1700 cm^–1^. The former are associated with C–H stretches, while the latter are combinations of C–C vibrations in the catalyst. None of these modes is involved in hydrogen bonding. In agreement with the experiment, we thus find that they are not affected by complexation.

We end this section with a discussion of the implications of the present study on conformational distributions observed under molecular beam conditions. For the bare catalyst, our studies clearly indicate that **sa1** is predominant, in perfect agreement with the calculated conformational energies. Intuitively, one might therefore expect that in Cat···Nitro complexes, the catalyst would also be present in the **sa1** conformation. However, Cat···Nitro complexes are generated and cooled under high-collision rate conditions with Ar. It can therefore very well be, as is indeed confirmed here, that during the three- and higher-body collisions needed to form the complex, conformational transitions of the catalyst occur that lead to lower-energy complexes. This observation therefore suggests that also larger complexes involving more than one reactant might be trapped under conditions as used here. Such exciting experiments would bring studies on the detailed structure of neutral reactive intermediates of catalytic reactions within reach.

In this work, we have employed molecular beam experiments in combination with infrared spectroscopy and quantum chemical calculations to investigate in detail the preferred conformation of the famous Takemoto organocatalyst and its coordination to the 1-(2-nitroethyl)­naphthalene nitroolefin, as part of the well-known Michael addition reaction. We show that this methodology provides a valuable assignment of the conformation of the catalyst and the geometry of its complex with the nitroolefin, both of which have been the center of significant experimental and computational efforts in the past. Calculations by themselves cannot address these questions, given that their outcome highly depends on the employed level of theory, something that is also expected for reaction pathways and transition states. Moreover, gas-phase infrared spectroscopy provides direct information about the intermolecular interactions in the complex, which for this catalyst involves primarily hydrogen bonding. These interactions manifest themselves most clearly in red-shifts observed for the vibrational modes involved in the hydrogen bonds. The methodology presented in this paper is broadly applicable and is thus expected to allow for further studies of other catalytic reactions, as well.

## Supplementary Material



## Data Availability

Data for this article, including Figures 1, 2, 4, and 6, are available at 10.34973/aw0k-r868.

## Notes

Synthesis of 1-(2-nitroethyl)­naphthalene, experimental setup, measured and computed infrared spectra of the bare catalyst, Cartesian coordinates, experimental curves, Boltzmann-weighted IR spectra, computed IR spectra of bare nitroolefin with PBE0 and M06-2X, relative energies of the Cat···Nitro complex, computed IR spectra of the bare catalyst with PBE0, and comparison of the experiment and computations for the five lowest-energy conformers (PDF)

## ∇

A.K.L.: Chemical Sciences Division, Lawrence Berkeley National Laboratory, Berkeley, CA 94720

## References

[1] MacMillan D. W. C. (2008). The
advent and development of organocatalysis. Nature.

[2] Taylor M. S., Jacobsen E. M. (2006). Asymmetric catalysis
by chiral hydrogen-bond donors. Angew. Chem.,
Int. Ed..

[3] Ahrendt K. A., Borths C. J., MacMillan D. W. C. (2000). New strategies for organic catalysis:
the first highly enantioselective organocatalytic diels–alder
reaction. J. Am. Chem. Soc..

[4] Bagheri I., Mohammadi L., Zadsirjan V., Heravi M. M. (2021). Organocatalyzed
asymmetric mannich reaction: an update. ChemistrySelect.

[5] Pasuparthy S. D., Maiti B. (2022). Enantioselective organocatalytic
michael addition reactions catalyzed
by proline/prolinol/supported proline based organocatalysts: an overview. ChemistrySelect.

[6] Schreiner P. R. (2003). Metal-free
organocatalysis through explicit hydrogen bonding. Chem. Soc. Rev..

[7] Luchini G., Ascough D. M. H., Alegre-Requena J. V., Gouverneur V., Paton R. S. (2019). Data-mining the diaryl­(thio)­urea conformational landscape:
Understanding the contrasting behavior of ureas and thioureas with
quantum chemistry. Tetrahedron.

[8] Ehrhard A. A., Gunkel L., Jäger S., Sell A. C., Nagata Y., Hunger J. (2022). Elucidating Conformation
and Hydrogen-Bonding Motifs
of Reactive Thiourea Intermediates. ACS Catal..

[9] Madarász A., Dósa Z., Varga S., Soós T., Csámpai A., Pápai I. (2016). Thiourea derivatives as brønsted
acid organocatalysts. ACS Catal..

[10] Okino T., Hoashi Y., Takemoto Y. (2003). Enantioselective
michael reaction
of malonates to nitroolefins catalyzed by bifunctional organocatalysts. J. Am. Chem. Soc..

[11] Okino T., Hoashi Y., Furukawa T., Xu X., Takemoto Y. (2005). Enantio- and
diastereoselective michael reaction of 1,3-dicarbonyl compounds to
nitroolefins catalyzed by a bifunctional thiourea. J. Am. Chem. Soc..

[12] Lippert K. M., Hof K., Gerbig D., Ley D., Hausmann H., Guenther S., Schreiner P. R. (2012). Hydrogen-bonding thiourea organocatalysts: the privileged
3,5-Bis­(trifluoromethyl)­phenyl group. Eur. J.
Org. Chem..

[13] Gaunt M. J., Johansson C. C. C., McNally A., Vo N. T. (2007). Enantioselective
organocatalysis. Drug Discovery Today.

[14] Supady A., Hecht S., Baldauf C. (2017). About underappreciated
yet active
conformations of thiourea organocatalysts. Org.
Lett..

[15] Zhu J.-L., Zhang Y., Liu C., Zheng A.-M., Wang W. (2012). Insights into
the dual activation mechanism involving bifunctional. J. Org. Chem..

[16] Kreienborg N. M., Merten C. (2018). How do substrates bind to a bifunctional thiourea catalyst?
a vibrational CD study on carboxylic acid binding. Chem. - Eur. J..

[17] Kreienborg N. M., Pollok C. H., Merten C. (2016). Towards an observation of active
conformations in asymmetric catalysis: interaction-induced conformational
preferences of a chiral thiourea model compound. Chem. - Eur. J..

[18] Hamza A., Schubert G., Soós T., Pápai I. (2006). Theoretical
studies on the bifunctionality of chiral thiourea-based organocatalysts:
competing routes to C–C bond formation. J. Am. Chem. Soc..

[19] Gimeno M. C., Herrera R. P. (2016). Hydrogen bonding networks in chiral thiourea organocatalysts:
evidence on the importance of the aminoindanol moiety. Cryst. Growth Des..

[20] Izzo J. A., Myshchuk Y., Hirschi J. S., Vetticatt M. J. (2019). Transition
state analysis of an enantioselective Michael addition by a bifunctional
thiourea organocatalyst. Org. Biomol. Chem..

[21] Bakels S., Gaigeot M.-P., Rijs A. M. (2020). Gas-phase
infrared spectroscopy of
neutral peptides: insights from the far-IR and THz domain. Chem. Rev..

[22] Bakels S., Meijer L. M., Greuell M., Porskamp S. B. A., Rouwhorst G., Mahé J., Gaigeot M.-P., Rijs A. M. (2019). Interactions
of
aggregating peptides probed by IR-UV action spectroscopy. Faraday Discuss..

[23] Rijs A. M., Compagnon I., Oomens J., Hannam J. S., Leigh D. A., Buma W. J. (2009). Stiff, and Sticky in the Right Places: Binding Interactions
in Isolated Mechanically Interlocked Molecules Probed by Mid-Infrared
Spectroscopy. J. Am. Chem. Soc..

[24] Bakker D. J., Peters A., Yatsyna V., Zhaunerchyk V., Rijs A. M. (2016). Far-Infrared Signatures of Hydrogen
Bonding in Phenol
Derivatives. J. Phys. Chem. Lett..

[25] MacAleese L., Maître P. (2007). Infrared spectroscopy
of organometallic ions in the
gas phase: From model to real world complexes. Mass Spectrom. Rev..

[26] Holland M., Berden G., Oomens J., Meijer A. J. H. M., Schäfer M., Gilmour R. (2014). Infrared Multiphoton Dissociation
Spectroscopic Analysis of Noncovalent Interactions in Organocatalysis. Eur. J. Org. Chem..

[27] Paul M., Peckelsen K., Thomulka T., Martens J., Berden G., Oomens J., Neudörfl J.-M., Breugst M., Meijer A. J. H. M., Schäfer M., Berkessel A. (2021). Breslow Intermediates
(Amino Enols) and Their Keto Tautomers: First Gas-Phase Characterization
by IR Ion Spectroscopy. Chem. - Eur. J..

[28] Ferrari P., Lemmens A. K., Redlich R. (2024). Infrared bands
of neutral gas-phase
carbon clusters in a broad spectral range. Phys.
Chem. Chem. Phys..

[29] Yatsyna Y., Bakker D. J., Salén P., Feifel F., Rijs A. M., Zhaunerchyk V. (2016). Infrared Action Spectroscopy of Low-Temperature Neutral
Gas-Phase Molecules of Arbitrary Structure. Phys. Rev. Lett..

[30] Fielicke A. (2023). Probing the
binding and activation of small molecules by gas-phase transition
metal clusters via IR spectroscopy. Chem. Soc.
Rev..

[31] Neese F. (2022). Software update:
The ORCA program system—Version 5.0. Wiley Interdiscip. Rev.: Comput. Mol. Sci..

[32] de
Souza B. (2025). GOAT: A Global Optimization Algorithm for Molecules and Atomic Clusters. Angew. Chem., Int. Ed..

[33] Kreienborg N. M., Merten C. (2019). How to treat C–F stretching vibrations? A vibrational
CD study on chiral fluorinated molecules. Phys.
Chem. Chem. Phys..

[34] van
Outersterp R. E., Houthuijs K. J., Berden G., Engelke U. F., Kluijtmans L. A. J., Wevers R. A., Coene K. L. M., Oomens J., Martens J. (2019). Reference-standard free metabolite identification using
infrared ion spectroscopy. Int. J. Mass Spectrom..

