# Supplementary material for: Light on Catalytic Reaction Mechanisms: Uncovering the Conformation of Thiourea-Based Organocatalysts and Their Interaction with Nitroolefins Using Mid-infrared Spectroscopy
Source: J Phys Chem Lett. 2025 Jun 11;16(24):6178–84. doi: 10.1021/acs.jpclett.5c01093 (PMC12183758; doi:10.1021/acs.jpclett.5c01093)
Supplement: Supplementary file 1 [file jz5c01093_si_001.pdf]

Supporting information

Light on catalytic reaction mechanisms: uncovering the conformation of thiourea-based organo-catalysts and their interaction with nitroolefins using mid-infrared spectroscopy

Piero Ferrari[+,*], Alexander K. Lemmens[+], Wybren Jan Buma[+,‡*]

[+] HFML-FELIX, Radboud University, Nijmegen 6525 ED, The Netherlands

[‡] Molecular Photonics, Van 't Hoff Institute for Molecular Sciences, Faculty of Science, University of Amsterdam, 1090 GD Amsterdam, The Netherlands

Content

1. Synthesis of 1-(2-nitroethyl)naphthalene
2. Experimental setup
3. Measured and computed infrared spectra of the bare catalyst
4. Cartesian coordinates
5. Experimental curves
6. Boltzmann-weighted IR spectra
7. Computed IR spectra of bare nitroolefin with PBE0 and M06-2X
8. Relative energies of the Cat··Nitro complex
9. Computed IR spectra of the bare catalyst with PBE0
10. Comparison of experiment and computations for the 5 lowest-energy conformers

1. Synthesis of 1-(2-nitroethyl)naphthalene

All experiments were performed under a dry nitrogen atmosphere, unless stated otherwise.

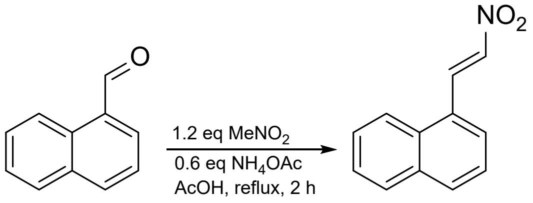

To a solution of the aldehyde in glacial acetic acid 1.2 equivalents of nitromethane and 0.6 equivalent of ammonium acetate were added. The resulting mixture was stirred at reflux temperature for 2 hours. The reaction mixture was then poured in water and extracted three times with dichloromethane. The organic layers were washed with brine, dried (Na_2SO_4) and concentrated in vacuo. The crude product was flash chromatographed over silica using a gradient of a mixture of ethyl acetate and hexanes to afford the product in a yield of 69 %. ^{1}H-NMR (400 MHz, $CDCl_3$) d 8.85 (d, J = 13.4 Hz, 1H) 8.14 (d, J = 8.4 Hz, 1H) 8.01 (d, J = 8.2 Hz, 1H) 7.93 (d, J = 8.0 Hz, 1H) 7.77 (d, J = 7.2 Hz, 1H) 7.67 (d, J = 13.4 Hz, 1H) 7.66 (m , 1H) 7.60 (t, J = 7.7 Hz, 1H) 7.53 (t, J = 7.7 Hz, 1H) ; TLC : $R_f \approx 0.4$ (ethyl acetate/petroleum ether 40-65 °C – 5/95).

2. Experimental setup

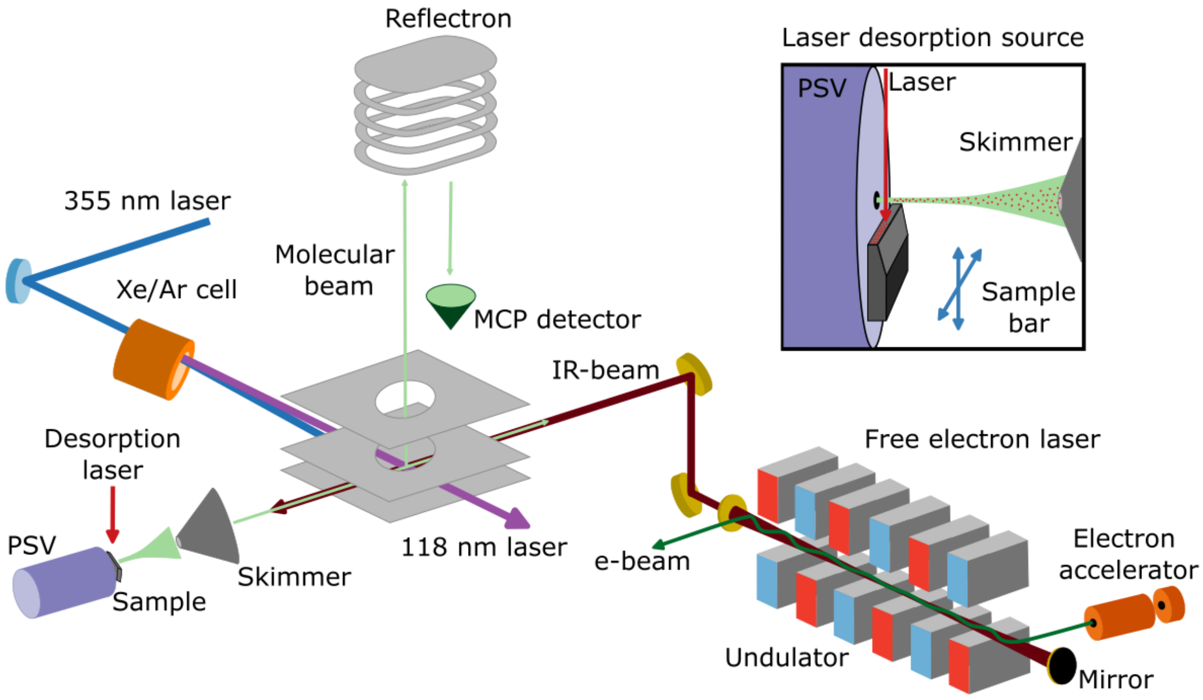

Figure S1. Schematic representation of the molecular beam setup at HFML-FELIX (Nijmegen, The Netherlands), including a pulsed gas valve (PSV) and a laser desorption source, together with a reflectron time-of-flight mass spectrometer. A Xe/Ar cell pumped with the third harmonic of a Nd:YAG laser (355 nm) is used to generate 118 nm laser light, which ionizes the species in the molecular beam. Within the same interaction volume, the counter-propagating infrared laser light of the free electron laser FELIX resonantly excites vibrational modes of the molecules.

3. Measured and computed infrared spectra of the bare catalyst

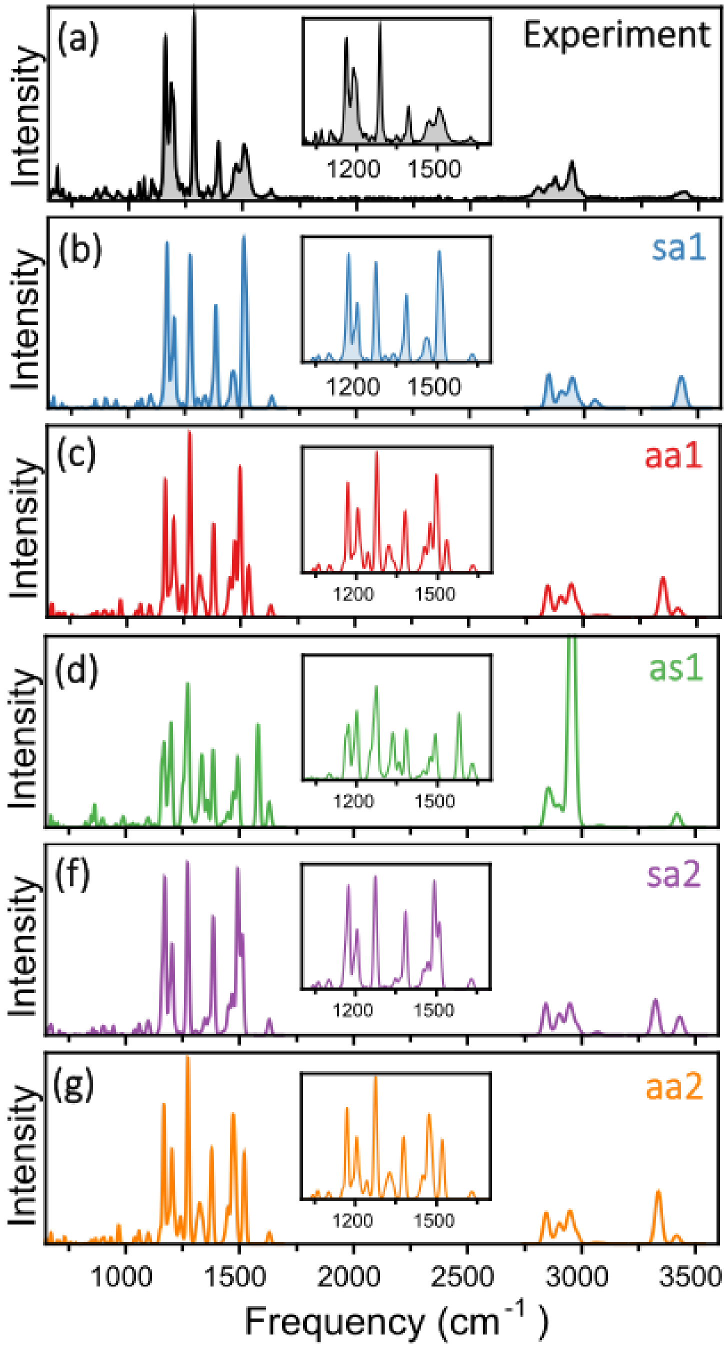

Figure S2. (a) Measured infrared spectrum of Takemoto's catalyst. (b-g) Computed infrared spectra of the different conformers of the bare catalyst.

4. Cartesian coordinates

Below are given the XYZ coordinates of the species discussed in the main article. Numbers are given in Å.

sa1

C	3.20229359464990	0.71710115893314	0.57858037453729
C	4.69418535648574	1.07514195185936	0.55182697359637
C	5.48226083848814	0.20208019626492	1.52921491171991
C	5.27520603594654	-1.28284578974359	1.24272907556547
C	3.79131132118768	-1.63787347397254	1.23453038107001
C	3.04879460233950	-0.76488684415845	0.22428335412143
H	6.54270283722159	0.45575414287892	1.48354148482888
H	5.08446013456386	0.93176050709038	-0.46063844858671
H	4.82819434070315	2.12838777194592	0.80362911135058
H	2.85377017717390	0.84202166560280	1.61361343128866
H	5.70416486025763	-1.52861273288788	0.26593622369923
H	5.80198384374423	-1.89050515296716	1.97998428348316
H	3.63912148371599	-2.68437753831123	0.97009070942176
H	3.35922543848049	-1.47645482159235	2.22776173680267
H	3.48044905167481	-0.95661152542381	-0.76215932566355
H	5.14956753188725	0.42073637542044	2.54945571138132
C	1.12423823883955	-2.04804858909945	-0.63757832850886
C	-1.20782336931960	-1.13143812623381	-0.41709138567500
C	-0.96383963211589	0.23518674666503	-0.53303037467700
C	-2.48610897688119	-1.56394004887985	-0.04775916463386
C	-1.98168257018966	1.13811118986498	-0.25148981858190
H	0.00218761854178	0.60804572319376	-0.85301978298693
C	-3.48441827651244	-0.64666922408815	0.20447818874528
H	-2.68453821741346	-2.62357495132302	0.04379824385425
C	-3.24544095627845	0.72023326765590	0.11712545927093
H	-4.03007345447618	1.43481839939218	0.32482520219350
C	2.53155314497080	1.45868480512615	-1.66820834506349
H	2.41319970938806	0.42879343206502	-2.00924607469184
H	1.77987220269971	2.06683618777146	-2.17398404579204
H	3.52064118331184	1.81932867690043	-1.98474986009245
C	2.30848598765585	2.94285107950323	0.19963284663286
H	2.14408894825368	2.99186207597352	1.27699760443972
H	3.24208046147538	3.47383338827282	-0.03480312605082
H	1.49107919723955	3.46908803089429	-0.29271095088419
N	-0.25075581481199	-2.10605318921965	-0.71274648855759
H	-0.57837317957421	-2.95667999416712	-1.14399323821139
N	1.63479580040899	-1.08697714512443	0.13771575195784
H	1.01136991388093	-0.44386567414193	0.60225063175547
N	2.31690485923336	1.55586692152488	-0.23324899408303
S	2.02922086971873	-3.17120709458946	-1.47168718125662
C	-1.66699801250161	2.60744740354483	-0.33328125146933
C	-4.86601383656038	-1.10739235161859	0.58663903132473
F	-2.76711891343576	3.35320324630498	-0.44339571284664
F	-0.88445096835903	2.88733094788515	-1.38338540044454
F	-1.01264667459582	3.02616850826412	0.75888681999139
F	-5.26444763588122	-0.54804374882575	1.73460437802096
F	-4.93352502660535	-2.43103324512899	0.74193556689811
F	-5.76612006862642	-0.76870553930143	-0.34375818919396

sa2

C	3.29445571279561	0.70547282343677	0.54851043902529
C	4.79802166995285	0.87086552829697	0.79364182970494
C	5.27865928273057	-0.11145225457328	1.86247034177695
C	4.93460573910066	-1.55114646190060	1.48711205477477
C	3.44469035542053	-1.70731591419837	1.19379904599411
C	3.02268526439885	-0.73400514549548	0.09492603742592
H	6.35392711066722	-0.00319416423201	2.01427539140332
H	5.34100963910798	0.68766772821704	-0.13870979689974
H	5.01560743588111	1.89646028155507	1.09863734485837
H	2.78370177814178	0.83719660191181	1.51167149984176
H	5.50340905759766	-1.84108989847538	0.59780342365037
H	5.23070799077536	-2.23295022862587	2.28567467311704
H	3.21511572982187	-2.72471895195637	0.87915067442368
H	2.85843595226881	-1.49419194617430	2.09386972371647
H	3.60970203926321	-0.96255946751385	-0.80143704753605
H	4.79878061757381	0.13629924355073	2.81507368170314
C	1.11046418841677	-1.80118076979965	-1.03696155531886
C	-1.23375782173889	-0.91107980398227	-0.76232547866791
C	-1.21343691545076	-0.49301825897917	0.56667021692938
C	-2.34290480046062	-0.59256866426405	-1.55351060200154
C	-2.27058326174379	0.25329842197942	1.06926013042311
H	-0.39610395587795	-0.77107271888335	1.21617364413042
C	-3.39007061099333	0.13332290572897	-1.02394912676692
H	-2.37353234034883	-0.91577079883736	-2.58586517722078
C	-3.36579159397385	0.57840879100847	0.29183907737943
H	-4.18534042330602	1.15531746506275	0.69744973306172
C	3.21867534183630	1.68196372110689	-1.70086890268571
H	3.19274441755351	0.68168003612964	-2.13406658782778
H	2.60673252979824	2.33277699303546	-2.32602137701329
H	4.25251825045382	2.05367032091950	-1.73873601408160
C	2.60934369302003	3.01082027576927	0.19825367297050
H	2.17752641621176	2.97900026448415	1.19921340575817
H	3.59326607556895	3.49825436389954	0.25889164730917
H	1.96923499858373	3.63133901862697	-0.42994065305087
N	-0.23071796493905	-1.69514791057228	-1.33685421635937
H	-0.51135203986188	-2.33448821702722	-2.06475465208780
N	1.61132834203321	-0.84321128627447	-0.24130984462117
H	1.13092796054464	0.05188117612332	-0.22625781559430
N	2.66910223261831	1.67074166686824	-0.35522660317294
S	1.98700735528603	-3.04750499712101	-1.70470616419782
C	-2.19313395145616	0.73545939174178	2.49353821102596
C	-4.60305727491106	0.44254688167504	-1.86051475475489
F	-3.39723239933435	1.02462492364203	2.99011182054052
F	-1.63335663435617	-0.17555343974246	3.29556587331017
F	-1.44827845150228	1.84630660142698	2.59046361157551
F	-4.94609287970778	1.73154046050737	-1.76556888631995
F	-4.40868345264530	0.17261567659295	-3.15311910137742
F	-5.66186740481515	-0.27165726466835	-1.46008284827350

as1

C	3.80886227304493	0.68477665584599	0.11245483418745
C	4.85200721090840	1.09633070610579	-0.93295956738818
C	6.06909102939980	0.18021477145508	-0.92720188034651
C	5.62873395602417	-1.25261420061495	-1.19938706130268
C	4.61581143121915	-1.69903654381846	-0.15489822640150
C	3.38849740155878	-0.78609907343999	-0.05707681778565
H	6.78960657906104	0.51147417463255	-1.67639415003880
H	4.40326420883187	1.05573578636435	-1.92978967565725
H	5.14333791339008	2.13508244010989	-0.75946001107646
H	4.26821324962842	0.75146571234476	1.10403502852940
H	5.18236001053575	-1.31167706690999	-2.19706794893346
H	6.48269242888305	-1.93137517633770	-1.19375633349045
H	4.27445229316150	-2.71759111312638	-0.34866992890837
H	5.10476952587824	-1.70531160918696	0.82681694938660
H	2.79578499838790	-0.88208499629236	-0.97475288844293
H	6.57148057777710	0.23042074518697	0.04410613129442
C	1.22916327686261	-1.39162205000727	1.11328407665290
C	-0.75940933632680	-0.20704016419035	0.24148879323332
C	-1.58504117020978	-1.26191495684061	-0.15593379155540
C	-1.29342323669420	1.06947839960785	0.35095226507145
C	-2.91462403753189	-1.01549748910798	-0.43023612068820
H	-1.17758285954653	-2.25626700096984	-0.25459215443087
C	-2.63440276025775	1.29420233656301	0.06626314342262
H	-0.66220168322859	1.88923001582147	0.67471124969519
C	-3.46135290326949	0.25981204437156	-0.32189634385264
H	-4.50436618995671	0.43558414690264	-0.54479291745061
C	2.14739886292050	1.98583808998722	-1.17200383982574
H	2.01701907183079	1.11363361581956	-1.81389326828263
H	1.17166519482709	2.46169337304819	-1.05745724029840
H	2.81107248861450	2.69719113362789	-1.67765381667532
C	2.84086378630636	2.74232208432021	0.99621325333692
H	3.08255673168228	2.41810757690788	2.00750708910566
H	3.64382652402167	3.39804843733491	0.63147476740852
H	1.92246711895251	3.32948896857771	1.03484893759781
N	0.61005881944933	-0.37266138109229	0.45610553517261
H	1.20217704162523	0.47009545131899	0.38963505558824
N	2.59062975368955	-1.29218390983823	1.06629442622871
H	3.02477449315404	-2.03601880952593	1.59245629821501
N	2.63633681891318	1.58147748607360	0.14257877722325
S	0.48329855207469	-2.61132996588065	1.95582699195984
C	-3.81613963175960	-2.14397892591838	-0.85138457277302
C	-3.15409624912485	2.69971609341169	0.17704044527387
F	-4.63046899717586	-2.51973771725343	0.14196821017600
F	-3.13207345049244	-3.22379898111028	-1.23340007851787
F	-4.59923130418879	-1.78386719398570	-1.87730410571057
F	-2.89235173588979	3.23009446263278	1.37901838891790
F	-4.47136711275759	2.77294586911350	-0.01399528886036
F	-2.57569196420389	3.50591274796166	-0.72740261898382

aa1

C	-4.68097579259194	0.54795885513162	-0.37289078933621
C	-5.99195466727091	0.15566291904477	0.31714226164339
C	-6.46016181392557	-1.21883440465750	-0.16290161275274
C	-5.38326889708194	-2.28032878374499	0.05047053172404
C	-4.06236372073002	-1.86792300673355	-0.59395398718151
C	-3.61984692677773	-0.51110503789500	-0.05038291390639
H	-7.38072185117902	-1.50046103381106	0.35110768871984
H	-5.84040904589512	0.13120724391688	1.40064506353757
H	-6.75979707754909	0.90490211733993	0.11430894357899
H	-4.85301480353071	0.51080185610633	-1.45708373395270
H	-5.22360671872987	-2.42543631002534	1.12377481540409
H	-5.71193446885789	-3.24011357352697	-0.35077551757602
H	-3.28418765100563	-2.60301648014107	-0.39193199456198
H	-4.17715553537094	-1.79152792944319	-1.68000637558963
H	-3.51008091184232	-0.60609154766349	1.03528452306899
H	-6.70003212295630	-1.15970653904935	-1.22978406754113
C	-1.14751337483156	-0.45522863262697	-0.11860553502569
C	1.26357398067282	0.15614638527525	-0.40257365255840
C	1.90551008376995	-1.07793964856745	-0.28334362672293
C	2.00895265567705	1.32426666166909	-0.32366504170828
C	3.26811861473889	-1.11107014130331	-0.06385616842401
H	1.33929472091982	-1.99185612891248	-0.36222570191170
C	3.38117197800515	1.26506076089633	-0.11910786639210
H	1.51770416945368	2.28634400774843	-0.40495105074309
C	4.02484595886380	0.05218243591279	0.02287039116962
H	5.09147335176549	0.00652744319495	0.19638054570898
C	-3.84078544975121	2.15573072351175	1.28230647961635
H	-3.11526616287200	1.42594967166470	1.64308527446535
H	-3.38618968868704	3.14423597567793	1.35584178148895
H	-4.71546713523226	2.13894729634678	1.94762661599034
C	-5.04432120598656	2.93596224453853	-0.63725749428454
H	-5.29102233194902	2.71695767152054	-1.67695099858033
H	-5.98286699983908	3.04614851433505	-0.07539225873604
H	-4.52269783478589	3.89333193490327	-0.60262020053809
N	-0.11042442532160	0.26421939033423	-0.66683908583811
H	-0.36843760893972	1.07781074967470	-1.20499804176107
N	-2.34826795185503	-0.06554974833118	-0.59527432954974
H	-2.41115914351012	0.84579635303926	-1.03502425948589
N	-4.17914832937665	1.89804144161508	-0.10817580312147
S	-0.95517776302672	-1.64482709358378	1.02522163651849
C	3.98338219757675	-2.42799537955447	0.07232094684836
C	4.16186131730346	2.54939664854041	-0.08635933149291
F	4.93923693262803	-2.55760768247754	-0.85846728205908
F	3.16235495778155	-3.46969139854830	-0.05295576587445
F	4.58930573696247	-2.53277921995395	1.26137155363093
F	5.34999904117025	2.40130128075837	0.50100036447048
F	3.50360648135553	3.51239834311673	0.57105807071492
F	4.38788823261495	3.01486179473720	-1.32325700109343

aa2

C	-4.67822145714290	0.59294471447498	-0.29211308599395
C	-6.01379303261298	0.13067633120496	0.30058521028422
C	-6.45286584885353	-1.19020855963925	-0.33272979115154
C	-5.37916847774475	-2.26513138309400	-0.17909682536359
C	-4.03538430820811	-1.78797079123172	-0.72357551503497
C	-3.62303125765768	-0.48879777521926	-0.03476715131520
H	-7.39271567880575	-1.52328939749723	0.11073468496681
H	-5.90639513773731	-0.00182529460728	1.38154601795348
H	-6.77716029304849	0.89511668738580	0.14249749274249
H	-4.80532374002117	0.66453686754576	-1.38067150509676
H	-5.26433234903549	-2.51534846696699	0.88047346261162
H	-5.68412316351526	-3.18128096359537	-0.68692789470167
H	-3.26184916574670	-2.53820327065468	-0.56253893681991
H	-4.10570318024011	-1.60661199099024	-1.80096850580468
H	-3.55122999015636	-0.68788225835047	1.04012042665099
H	-6.64723019375358	-1.02608820016665	-1.39789198180388
C	-1.15034815654184	-0.44500965901933	-0.03465835625589
C	1.27191228178192	0.10179173960829	-0.31057780705514
C	1.75272519458518	-0.00601002616664	0.99474830969263
C	2.16872972287030	0.12394454792074	-1.37017045147997
C	3.11324612526131	-0.10868319943627	1.20825319301628
H	1.06423318012906	-0.01196194079332	1.82510550537780
C	3.53291501884058	0.03161804652038	-1.13047684100454
H	1.80411822513941	0.19256891295648	-2.38771332569187
C	4.02092870930614	-0.09204153812325	0.15576252052372
H	5.08296360218078	-0.18021227952518	0.34008386268554
C	-3.93133999992344	2.03141982548731	1.55212516106076
H	-3.22424609923871	1.26806422157304	1.87813436421790
H	-3.48230914624344	3.00636417167463	1.74390003431240
H	-4.83986161013386	1.95337366924359	2.16590791344969
C	-5.03503616128890	2.99596930425108	-0.34313580831921
H	-5.22288369113754	2.88158660650335	-1.41146973313445
H	-6.00379089219369	3.05111173894822	0.17398521367911
H	-4.51984218574907	3.94427385509184	-0.18516998018606
N	-0.09480310732943	0.25422499816250	-0.58484924172184
H	-0.30271081330620	0.73253649676352	-1.44878229201856
N	-2.33729199909766	0.01045743577304	-0.49254029263893
H	-2.37495140296116	0.98289529896381	-0.78141812124753
N	-4.19614751782659	1.90968859077686	0.12758494471821
S	-0.98155093101551	-1.70190799674403	1.03307047915021
C	3.65227504547622	-0.18611196659440	2.61119913828899
C	4.48331597341075	0.10201054571621	-2.29380979623184
F	4.22578538649432	0.97112866701166	2.97231571050183
F	2.70246439993741	-0.45278433449659	3.50757121043821
F	4.59191241488700	-1.13164415904972	2.72408575867755
F	5.64879871937617	-0.48972536437987	-2.02565252647121
F	3.97533576224392	-0.48077592569182	-3.38585193705075
F	4.75492322634681	1.37194946847552	-2.62677891140647

Nitroolefin

C	-3.20447496030814	1.20604150569467	-0.02247814968205
C	-4.40574168816035	0.55670311743923	-0.11833236841894
C	-4.46335098009449	-0.84808664342576	-0.11837944255587
C	-3.31019327948113	-1.57524619570581	-0.02695239888109
C	-2.05477894573057	-0.93585229641442	0.07493604093596
C	-1.99363846248175	0.48671537355408	0.08910234373749
C	-0.71468792065294	1.11842445352356	0.19190829119785
C	0.42056429919718	0.33751362287006	0.27233945757444
C	0.35177907417693	-1.06081274433669	0.25110501057664
C	-0.86195160320544	-1.68473987983855	0.15639128678166
C	-0.61499476207944	2.56843752631162	0.24701553678280
C	0.43458838119872	3.28200601831958	-0.15293730701789
N	0.44207188807304	4.72073564304186	-0.00752722383086
O	-0.52346019779549	5.27600832778274	0.47601566469823
O	1.44802538001760	5.28329200530645	-0.39672955328949
H	1.33821765047784	2.91555086199019	-0.61561927386292
H	-1.44838983128436	3.13646607700229	0.64661743793317
H	1.38554123457392	0.81603803033635	0.39237039772563
H	1.26310172779487	-1.64210630337050	0.32491756365130
H	-0.92545596052023	-2.76764868794545	0.14763577904841
H	-3.33973810685249	-2.65961724029738	-0.03452578252048
H	-5.42121756865589	-1.34881916990779	-0.19631690131826
H	-5.32107268800615	1.13131769310971	-0.19987118446109
H	-3.19039168020124	2.28830290495993	-0.04607722480463

Iso1

C	1.17524663181090	-1.88389668415381	1.02135358064246
C	1.69493291989623	-1.68506752819798	2.45000558358533
C	3.19570623848861	-1.43526512815091	2.48294813711427
C	3.55829328886968	-0.22682308546013	1.63502548586526
C	3.04047752317965	-0.38846179674846	0.21476284409346
C	1.53506453484139	-0.65511328341975	0.17867012225785
H	3.53066429181523	-1.29481605060406	3.51477081044549
H	1.18110501948612	-0.83204444945467	2.90758262112838
H	1.44581799367833	-2.56270655495118	3.05301868345410
H	1.71299279220536	-2.73855899089536	0.58888839623956
H	3.12081616177900	0.67477212949556	2.08031964824794
H	4.64098173362962	-0.07374976186116	1.62099173261383
H	3.24709824861539	0.50760327912207	-0.37723346388559
H	3.55952667030074	-1.22363746677471	-0.27249515944191
H	1.01550373749557	0.22637777022807	0.56325985365643
H	3.72203627507334	-2.31901843507464	2.10182741736730
C	0.42826314907258	0.10502983976961	-1.89178535960023
C	0.81682143215706	-1.08199191357478	-4.07490666782468
C	2.17133177267519	-1.39381386791210	-3.96398999642380
C	0.08488454988060	-1.62351476319968	-5.12937557306144
C	2.76026545755962	-2.25153071396660	-4.87697029310810
H	2.76784464192713	-0.95875330399295	-3.17367103236870
C	0.69490713221073	-2.46492250721165	-6.04440536069654
H	-0.96876930866728	-1.38770341387171	-5.22592849804244
C	2.03441709126690	-2.79721009121399	-5.92429539088052
H	2.50342001539284	-3.46902645519755	-6.63003930522208
C	-1.15050880337149	-1.27375411331443	1.52491934742542
H	-0.97503775102293	-0.26961360115839	1.13485096005539
H	-2.17571361653031	-1.54875617108408	1.26661877851570
H	-1.08458817774359	-1.24194799283811	2.62461265689922
C	-0.52130508732009	-3.57142816678959	1.36758529496695
H	-0.40565620701217	-3.71292888029397	2.45527671532156
H	-1.55406815007242	-3.83073238320178	1.11863311120133
H	0.13969374140920	-4.27788094281504	0.85984049468231
N	0.17128588708806	-0.19603789787097	-3.20903381634520
H	-0.49802522216356	0.43707863436473	-3.61770520121842
N	1.09245065104426	-0.81564983404558	-1.19167219090640
H	1.21951457860543	-1.73557307052424	-1.59720311999989
N	-0.24167105442922	-2.22587219954392	0.91944141370306
S	-0.15552883796795	1.54969840388378	-1.28751931647430
C	4.23467150739228	-2.53570755515529	-4.76912821864414
C	-0.13930756257330	-3.07932857996628	-7.13403879680212
F	4.54902166931633	-3.73495791414220	-5.26338305720941
F	4.66074396619064	-2.49318820118841	-3.50300348999481
F	4.95113560901139	-1.62845425764278	-5.45311112592439
F	0.60089518536977	-3.48312498151853	-8.16854387548166
F	-1.05491474602660	-2.22274098800616	-7.60191401622936
F	-0.80924667198699	-4.15516890454755	-6.68546168090645
O	1.27301448238489	-3.84573162925482	-1.81417701505180
O	1.45496271157100	-5.44266878653783	-3.24481629089632
N	0.79800383503765	-4.64443741829894	-2.60948110313672
C	-0.62143617153327	-4.66414560271521	-2.83638840961546
C	-1.44440517864466	-3.87367210584454	-2.14474503146431
C	-2.89000440293664	-3.86770700402287	-2.30497712312987
C	-3.55261910401140	-5.01379171276885	-2.69972330928962
C	-4.94311809283007	-5.03906956229315	-2.85708813935174
C	-5.67840312426897	-3.90895852264705	-2.62221409229964
C	-5.05463629822199	-2.71074550904725	-2.21596329252343
C	-3.64093093817578	-2.67755182818011	-2.04391815275088
C	-3.04609249992787	-1.45808007425941	-1.65209376589800

C	-3.80407598805082	-0.33798233756970	-1.43656110009382
C	-5.19965936852865	-0.37585769071270	-1.60446123548575
C	-5.80882793490520	-1.53896054083055	-1.98432762190770
H	-1.97476000465193	-1.39399127149736	-1.51157478609466
H	-3.31306052669248	0.58195011340868	-1.13950706265981
H	-5.78756096854444	0.51831625718840	-1.43311649733012
H	-6.88439779267375	-1.58049539435846	-2.11978913442823
H	-6.75660961850148	-3.91957916580302	-2.74204203471365
H	-5.43090069171495	-5.95866717467577	-3.15735739223727
H	-2.98838834752372	-5.92542147247503	-2.85835006928692
H	-0.99461026203874	-3.19760889934909	-1.41977398626118
H	-0.89319261646389	-5.35995284278341	-3.61562803688255

Iso2

C	1.95854869350094	-1.38903739716646	2.31139961950428
C	2.65643155334117	-1.07090445111539	3.63649180485209
C	3.73889006488050	-0.01411970507286	3.45528329118977
C	3.17572997807171	1.24884134032765	2.81969927245289
C	2.45215751278019	0.93660300504667	1.51851693617201
C	1.35620797878086	-0.10290746766111	1.73870746230451
H	4.20504074190814	0.21515454200385	4.41819041148379
H	1.91555535050705	-0.71107450625811	4.35950339059472
H	3.08802888875743	-1.98517172420207	4.05446292371046
H	2.73180089859702	-1.72017313134855	1.60276621418957
H	2.47441763418310	1.72558726339778	3.51512505875038
H	3.97349761037093	1.97489719419774	2.63937669967673
H	1.99946310334282	1.83737233112977	1.09919281299843
H	3.16044241840056	0.54853237718242	0.77644963527881
H	0.63440287987021	0.32126580992157	2.44754716078142
H	4.53277260218980	-0.41831297554145	2.81461899352460
C	-0.38212541404096	0.30892278472685	0.03625283475578
C	-2.00881265227483	0.11140836793716	-1.86339482650292
C	-3.14294633040607	0.73077823150222	-1.33448597867548
C	-2.00917317091783	-0.24798873804553	-3.21039422718081
C	-4.23040196819492	0.98262595438647	-2.15297452191228
H	-3.16252233883786	1.00962402360148	-0.29185158210926
C	-3.11344297545742	0.00083816626095	-4.00811654964456
H	-1.13420389053912	-0.72667863682628	-3.63400271284486
C	-4.23595793345478	0.62549781509943	-3.49408838682851
H	-5.09667128263394	0.82349261178655	-4.11763887648498
C	-0.12689351784694	-2.28357826609345	3.24898021278943
H	-0.87334949151326	-3.05966240643733	3.06305466495153
H	0.15681305677255	-2.34516297423932	4.31266052795316
H	-0.60682753635236	-1.31851552273956	3.07854548800092
C	1.57589682535386	-3.77773134207959	2.48007333212887
H	0.81386529480493	-4.54189268795625	2.30668317147353
H	2.36003329334669	-3.91122758306386	1.73140483232561
H	2.01229069778299	-3.96718927690483	3.47590013769987
N	-0.89114797256949	-0.23762184200563	-1.11667507481179
H	-0.29546658979652	-0.92353381661826	-1.56774566732217
N	0.64223844159591	-0.41766203076330	0.52136510391271
H	0.71730930924618	-1.38574111390764	0.23037212868244
N	0.98229335370294	-2.47031039361418	2.34108559886715
S	-0.91835467823619	1.71681102492680	0.74340815705679
C	-5.42683182140596	1.69305817598310	-1.58246980324479
C	-3.07359899845922	-0.45237375778492	-5.43719158455465
F	-5.57419183566727	1.46598093117535	-0.27400431851559
F	-6.56350574979906	1.31243217195130	-2.18344404728374
F	-5.33203211956438	3.02188426361748	-1.74252481887248
F	-1.93525465041003	-0.09373407722334	-6.04770477029794
F	-4.08463934032308	0.03241410358041	-6.16013192743833
F	-3.13931035619373	-1.79886443341878	-5.52984056304839
O	0.53904590619681	-2.37137499052670	-2.75063423480362
O	1.44526878482303	-3.45404773309781	-1.12681311494868
N	1.03281421129735	-3.38222972824209	-2.26936459350302
C	1.13301665956484	-4.56677460918937	-3.06761225566382
C	0.63894052738712	-4.58108912094021	-4.30927015273013
C	0.67947209749349	-5.69381483335636	-5.23370518792519
C	1.62480645073644	-6.69284270996424	-5.08951083333737
C	1.70558892011532	-7.75946428058164	-5.98881506356270
C	0.83659574393260	-7.82798724960069	-7.04535679097487
C	-0.14737655962709	-6.83760064625918	-7.24269670440097
C	-0.24208173391464	-5.74854645123539	-6.32932897233314
C	-1.25726853254181	-4.79026487929302	-6.54253491187852

C	-2.11070803423997	-4.89066312183616	-7.60844778638204
C	-2.00527627396278	-5.95968537808301	-8.51518004859158
C	-1.04463672101243	-6.91350326847678	-8.33115175577490
H	-1.38739410489622	-3.96353778991142	-5.85638554387274
H	-2.87756422355757	-4.13776308415808	-7.74813897235047
H	-2.68917530774347	-6.02584078698896	-9.35303122829940
H	-0.95665660340740	-7.74873168337017	-9.01761472843098
H	0.89660303788335	-8.64847174548795	-7.75257868005653
H	2.46496814267215	-8.51987453209740	-5.85261620789001
H	2.34559030458897	-6.63133683317483	-4.28249532333281
H	0.16502465519837	-3.66172209639019	-4.63355929095777
H	1.59571608582034	-5.38040667939185	-2.53128325848642

5. Experimental curves

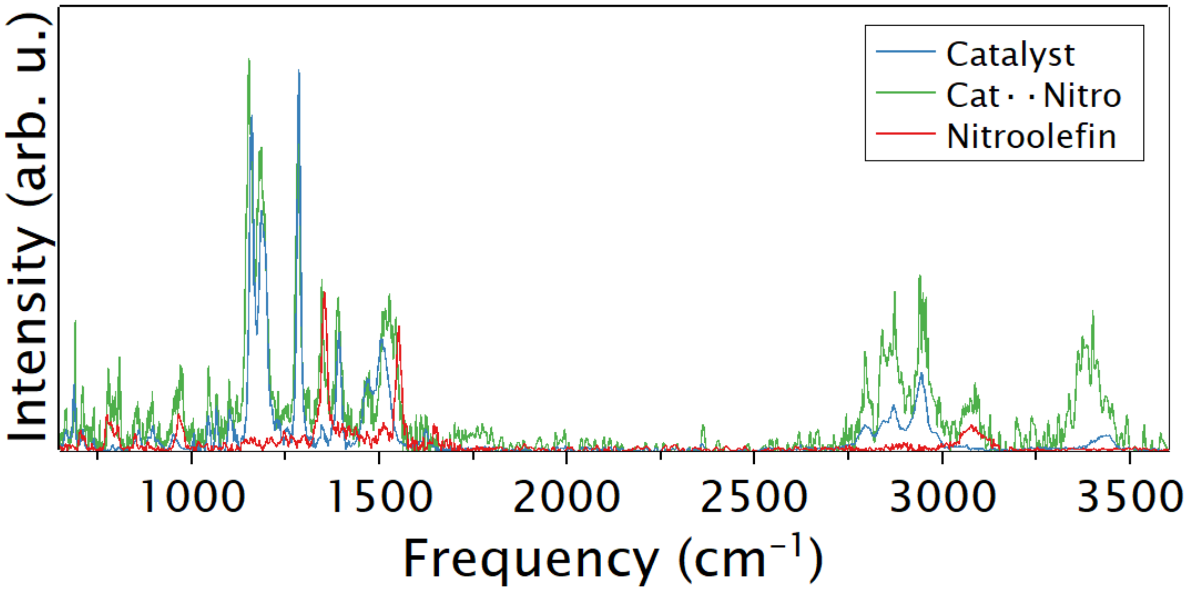

Figure S3. Measured infrared spectra of the bare catalyst (blue), nitroolefin (red) and Cat··Nitro complex (green).

6. Boltzmann-weighted IR spectra

Here we show that the infrared spectra of the bare catalyst and the Cat··Nitro complex constructed from the Boltzmann-weighted spectra of all the considered conformers only show very minor differences with the spectrum computed for the lowest-energy conformer. As discussed in the main text, the population of the different conformers is based on M06-2X calculations of Gibbs free energies.

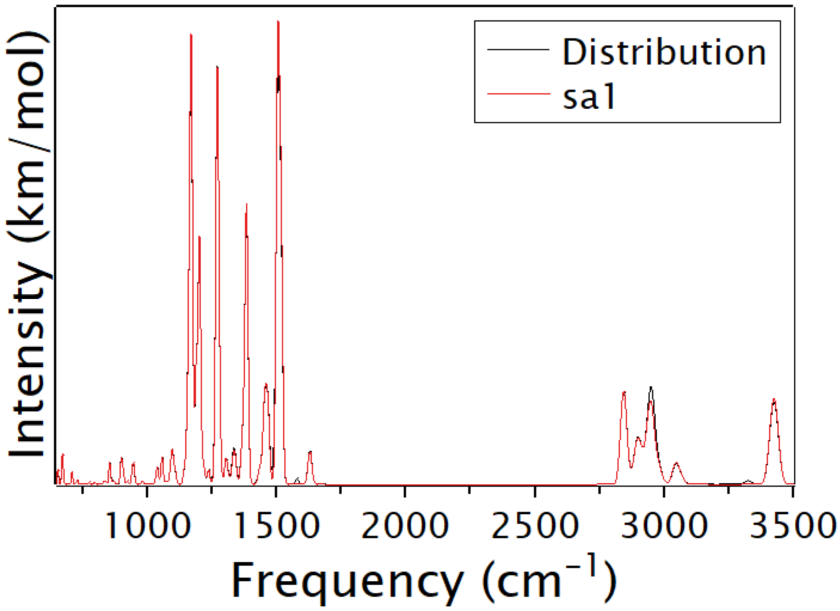

Figure S4. Comparison between the computed infrared spectrum of the bare catalyst calculated as a Boltzmann-weighted average of all conformers (black) and the spectrum of the dominant conformer **sa1**.

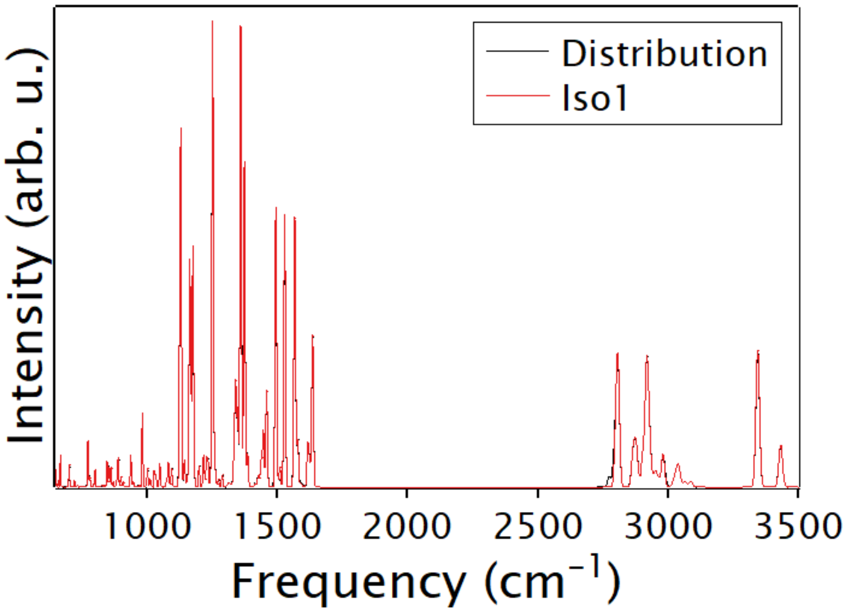

Figure S5. Comparison between the computed infrared spectrum of the Cat··Nitro complex calculated as a Boltzmann-weighted average of all conformers (black) and the spectrum of the dominant complex **Iso1**.

7. Computed IR spectra of 1-(2-nitroethyl)naphthalene with PBE0 and M06-2X

In the figure below we show that for the bare nitroolefin, PBE0 performs better than M06-2X. In the comparison that is presented, the scaling factor used for the bare catalyst is employed for the M06-2X calculations, whereas for PBE0 the scaling factor used for the **Cat··Nitro** complex is applied. Therefore, while M06-2X performs well for the bare catalyst, the strongest peaks of the nitroolefin in the range from 1200 and 1700 cm^{-1} are significantly blue shifted. In contrast, PBE0 predicts the positions of these peaks better, and performs also well for the **Cat··Nitro** complex. For the nitroolefin we notice, however, that the agreement between experiment and PBE0 calculations is still not perfect. As a result also in the **Cat··Nitro** complex some bands are shifted.

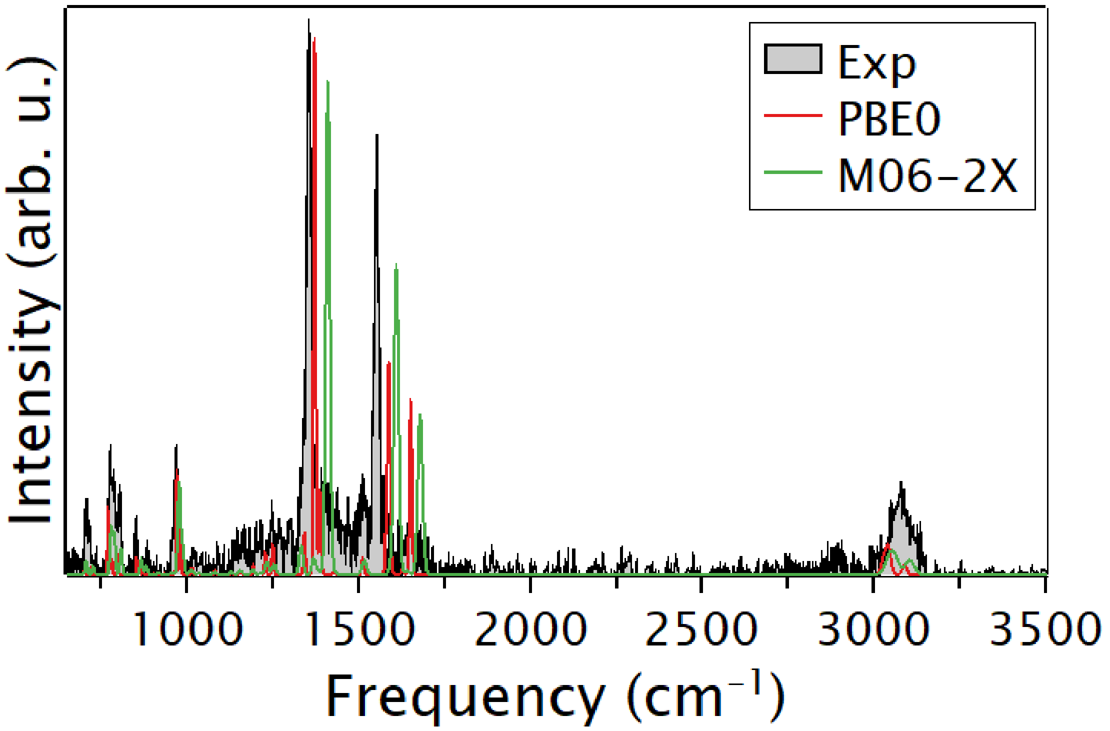

Figure S6. Comparison of the measured infrared spectrum of the bare nitroolefin (black) with calculations of the infrared absorption spectrum computed with the PBE0 (red) and M06-2X (green) functionals. For the PBE0 computations, the same scaling factor is employed for vibrational frequencies as for the **Cat··Nitro** complex, whereas for the M06-2X calculations the scaling factor used for the bare catalyst is employed.

8. Relative energies of the Cat··Nitro complex

Table S1. Relative energies (kJ/mol) of the lower-energy conformers of the **Cat··Nitro** complex, ordered based on the energies at the M06-2X level. Results employing the M06-2X and PBE0 functionals are presented. The third column corresponds to the similarity scores, as discussed in the main text. The two conformers discussed in the main manuscript are highlighted in red.

Conformer	M06-2X	PBE0	Similarity
1	0.0	8.6	0.42
2	9.5	6.0	0.36
3	12.6	8.2	0.14
4	12.8	10.9	0.24
5	12.9	8.3	0.20
6	12.9	8.2	0.25
7	14.1	4.2	0.33
8	16.8	3.7	0.34
9	17.2	7.5	0.33
10	17.4	8.4	0.33
11	17.6	5.4	0.33
12	18.4	7.5	0.27
13	18.8	5.7	0.32
14	19.8	13.1	0.22
15	20.4	8.6	0.27
16	20.8	20.0	0.19
17	20.8	5.8	0.33
18	21.0	10.3	0.22
19	21.2	17.9	0.23
20	22.7	19.8	0.18
21	23.1	22.6	0.15
22	23.5	10.0	0.29
23	24.3	4.4	0.33
24	24.6	9.8	0.33
25	24.7	16.7	0.30
26	27.0	16.0	0.36
27	28.9	6.4	0.34
28	28.9	9.0	0.29
29	29.1	10.8	0.34
30	30.9	21.2	0.25
31	32.2	23.9	0.31
32	32.8	4.0	0.28
33	35.5	21.6	0.29
34	38.1	0.2	0.28
35	38.1	0.1	0.32
36	43.1	5.8	0.30
37	46.0	0.4	0.30
38	46.1	0.0	0.30

Below we present the XYZ coordinates of the first 10 conformers on Table S1, as well as **Iso38**. Numbers in Å.

1

C	1.17524663181090	-1.88389668415381	1.02135358064246
C	1.69493291989623	-1.68506752819798	2.45000558358533
C	3.19570623848861	-1.43526512815091	2.48294813711427
C	3.55829328886968	-0.22682308546013	1.63502548586526
C	3.04047752317965	-0.38846179674846	0.21476284409346
C	1.53506453484139	-0.65511328341975	0.17867012225785
H	3.53066429181523	-1.29481605060406	3.51477081044549
H	1.18110501948612	-0.83204444945467	2.90758262112838
H	1.44581799367833	-2.56270655495118	3.05301868345410
H	1.71299279220536	-2.73855899089536	0.58888839623956
H	3.12081616177900	0.67477212949556	2.08031964824794
H	4.64098173362962	-0.07374976186116	1.62099173261383
H	3.24709824861539	0.50760327912207	-0.37723346388559
H	3.55952667030074	-1.22363746677471	-0.27249515944191
H	1.01550373749557	0.22637777022807	0.56325985365643
H	3.72203627507334	-2.31901843507464	2.10182741736730
C	0.42826314907258	0.10502983976961	-1.89178535960023
C	0.81682143215706	-1.08199191357478	-4.07490666782468
C	2.17133177267519	-1.39381386791210	-3.96398999642380
C	0.08488454988060	-1.62351476319968	-5.12937557306144
C	2.76026545755962	-2.25153071396660	-4.87697029310810
H	2.76784464192713	-0.95875330399295	-3.17367103236870
C	0.69490713221073	-2.46492250721165	-6.04440536069654
H	-0.96876930866728	-1.38770341387171	-5.22592849804244
C	2.03441709126690	-2.79721009121399	-5.92429539088052
H	2.50342001539284	-3.46902645519755	-6.63003930522208
C	-1.15050880337149	-1.27375411331443	1.52491934742542
H	-0.97503775102293	-0.26961360115839	1.13485096005539
H	-2.17571361653031	-1.54875617108408	1.26661877851570
H	-1.08458817774359	-1.24194799283811	2.62461265689922
C	-0.52130508732009	-3.57142816678959	1.36758529496695
H	-0.40565620701217	-3.71292888029397	2.45527671532156
H	-1.55406815007242	-3.83073238320178	1.11863311120133
H	0.13969374140920	-4.27788094281504	0.85984049468231
N	0.17128588708806	-0.19603789787097	-3.20903381634520
H	-0.49802522216356	0.43707863436473	-3.61770520121842
N	1.09245065104426	-0.81564983404558	-1.19167219090640
H	1.21951457860543	-1.73557307052424	-1.59720311999989
N	-0.24167105442922	-2.22587219954392	0.91944141370306
S	-0.15552883796795	1.54969840388378	-1.28751931647430
C	4.23467150739228	-2.53570755515529	-4.76912821864414
C	-0.13930756257330	-3.07932857996628	-7.13403879680212
F	4.54902166931633	-3.73495791414220	-5.26338305720941
F	4.66074396619064	-2.49318820118841	-3.50300348999481
F	4.95113560901139	-1.62845425764278	-5.45311112592439
F	0.60089518536977	-3.48312498151853	-8.16854387548166
F	-1.05491474602660	-2.22274098800616	-7.60191401622936
F	-0.80924667198699	-4.15516890454755	-6.68546168090645
O	1.27301448238489	-3.84573162925482	-1.81417701505180
O	1.45496271157100	-5.44266878653783	-3.24481629089632
N	0.79800383503765	-4.64443741829894	-2.60948110313672
C	-0.62143617153327	-4.66414560271521	-2.83638840961546
C	-1.44440517864466	-3.87367210584454	-2.14474503146431
C	-2.89000440293664	-3.86770700402287	-2.30497712312987
C	-3.55261910401140	-5.01379171276885	-2.69972330928962
C	-4.94311809283007	-5.03906956229315	-2.85708813935174
C	-5.67840312426897	-3.90895852264705	-2.62221409229964

C	-5.05463629822199	-2.71074550904725	-2.21596329252343
C	-3.64093093817578	-2.67755182818011	-2.04391815275088
C	-3.04609249992787	-1.45808007425941	-1.65209376589800
C	-3.80407598805082	-0.33798233756970	-1.43656110009382
C	-5.19965936852865	-0.37585769071270	-1.60446123548575
C	-5.80882793490520	-1.53896054083055	-1.98432762190770
H	-1.97476000465193	-1.39399127149736	-1.51157478609466
H	-3.31306052669248	0.58195011340868	-1.13950706265981
H	-5.78756096854444	0.51831625718840	-1.43311649733012
H	-6.88439779267375	-1.58049539435846	-2.11978913442823
H	-6.75660961850148	-3.91957916580302	-2.74204203471365
H	-5.43090069171495	-5.95866717467577	-3.15735739223727
H	-2.98838834752372	-5.92542147247503	-2.85835006928692
H	-0.99461026203874	-3.19760889934909	-1.41977398626118
H	-0.89319261646389	-5.35995284278341	-3.61562803688255

2

C	1.66786262274428	-1.82402776766772	1.34593771341953
C	2.60118909431048	-1.00109653739737	2.24050279840054
C	3.41648189314308	-0.00074162810982	1.43173574545023
C	2.51150807678311	0.92100585283113	0.62837508486882
C	1.55851646153334	0.12346917647041	-0.24884919972802
C	0.74682270879592	-0.87324376574899	0.57604947703723
H	4.06311398377266	0.58054981818723	2.09556858444660
H	2.00730380552408	-0.46243634195184	2.98793398763959
H	3.26727668219484	-1.67171174834950	2.79019682103452
H	2.29338421070365	-2.33575803138533	0.60002391180960
H	1.92991696848809	1.54895830505336	1.31403194227476
H	3.10506868670465	1.60189983939637	0.01179524374072
H	0.86273130081490	0.78675990060616	-0.76944734994296
H	2.12414721256096	-0.42682838490441	-1.01071092955422
H	0.12235247896595	-0.30540031618545	1.27175263530780
H	4.08042263076429	-0.54399749140019	0.74801278448514
C	-1.43087823859316	-1.31605766518655	-0.48717463315315
C	-1.96414146273630	-3.57860377353091	-1.45139862286669
C	-1.26854253103203	-4.41192117956535	-0.57532825935649
C	-2.54531119041853	-4.13128931959536	-2.59316412699973
C	-1.15412788453379	-5.76428931587486	-0.86339085292055
H	-0.82919162390418	-4.01664467810584	0.33558579419763
C	-2.42841091614870	-5.48365660485495	-2.85471965233220
H	-3.09144264137718	-3.49188961196121	-3.27745392295779
C	-1.72396485713767	-6.31582069445550	-1.99795773819151
H	-1.63093152025072	-7.37243758409164	-2.20561326003105
C	0.02784406072681	-2.45025902924701	3.06560035180229
H	-0.65595301709799	-1.67948749250070	2.70557507355322
H	-0.57733205126801	-3.30037370271289	3.38959432490554
H	0.55076546202416	-2.06132033037189	3.95476254651695
C	1.75886708223586	-3.97813605942948	2.44742951785667
H	2.38057201629360	-4.32375204569568	1.61870484743233
H	2.41973593349115	-3.72028894533061	3.29194031855977
H	1.13164241341579	-4.81370787001181	2.76538705209103
N	-2.16850354356171	-2.21772039868560	-1.21729166317228
H	-3.05016948045037	-1.83112841173877	-1.51520873078315
N	-0.16094419348374	-1.63606589336995	-0.25294751699456
H	0.23001522108382	-2.45000071698437	-0.70773499392345
N	0.91857861071894	-2.88500066325643	2.01132241082157
S	-2.16367564583154	0.10701573773537	-0.00561451599801
C	-0.38470419619558	-6.63560613536674	0.09330864099870
C	-3.01942419056326	-6.03429341580106	-4.12254897288064
F	-0.57786936401316	-7.93608465914272	-0.14383739758490

F	-0.74585280489147	-6.40551838095153	1.36465035039103
F	0.93187382987817	-6.40882475950470	0.01553243740005
F	-4.17188067752171	-5.43116924102339	-4.43842578668992
F	-3.26060748841239	-7.34454497674233	-4.03841323628024
F	-2.18989240938355	-5.85318724994429	-5.16475607935987
O	1.56026156794999	-3.14679529730594	-2.33885605809819
O	1.74093068123134	-4.97182178248712	-3.46785902980715
N	1.41036080616137	-3.80987691240757	-3.35463233188828
C	0.79923726924234	-3.20678012347452	-4.50998157317063
C	0.40358153036589	-1.93338482216499	-4.48170982348117
C	-0.19760619628802	-1.21186342264544	-5.58849300615701
C	0.06631077158998	-1.58475089017636	-6.89236189168403
C	-0.47915928412260	-0.88989803414613	-7.97716131297781
C	-1.29354381363524	0.18839600110390	-7.75807563132736
C	-1.59718924490619	0.61346812772772	-6.44793834101730
C	-1.04805040342812	-0.08810479998006	-5.33647538918052
C	-1.39579679402910	0.34778436054619	-4.03877186309041
C	-2.22222332646441	1.42295328217878	-3.84929747784031
C	-2.75190784147689	2.11981713489485	-4.94963730404560
C	-2.44521965535356	1.72013879099148	-6.22006608822878
H	-1.02223620037112	-0.17066917109101	-3.16503272960206
H	-2.46962376639523	1.72451254181909	-2.83782455836089
H	-3.40561727353168	2.96855226656410	-4.78618528446656
H	-2.85330864795877	2.24439127985959	-7.07769246565029
H	-1.71638868197839	0.73630549606578	-8.59354245820018
H	-0.24318779156292	-1.20318435990865	-8.98701920214503
H	0.74035023176487	-2.41189201287431	-7.08247048033318
H	0.55022728947053	-1.41078569079756	-3.54267326298543
H	0.69734225486012	-3.90656977443678	-5.32524639100199

3

C	3.10387591378085	-1.50935318115018	1.17735887066112
C	3.72052256335463	-2.39472762751095	2.26234926574965
C	3.46240975203302	-1.87713684961337	3.66658514465817
C	1.96598150071994	-1.77741076197998	3.90260345704046
C	1.33468378158097	-0.85272184114932	2.87959969992871
C	1.61094352597408	-1.23987010329800	1.42485570417413
H	3.92793960088783	-2.54282054627469	4.39858067466261
H	3.29856404853695	-3.40252664717688	2.19473382184740
H	4.79433432078316	-2.49261808137738	2.07467821641862
H	3.59940679727679	-0.53257858403124	1.20983920695445
H	1.51588628096885	-2.77419026631567	3.83228905193166
H	1.75143774493571	-1.40705232583448	4.90867994534389
H	0.25100213840466	-0.80241583595351	3.01697585022190
H	1.72631726692739	0.16139803683649	3.03828909253771
H	1.04280742029638	-2.14990027373299	1.19702871048748
H	3.92413558668991	-0.89106342503899	3.79946087950337
C	0.50593767073815	-0.15922890012685	-0.60863743828054
C	0.66357348876959	-1.36933087972181	-2.76197305865063
C	0.33880441196196	-0.37537754217191	-3.68260512899317
C	0.72193203185367	-2.69326392005946	-3.18694011987632
C	0.06369512417158	-0.72017599170851	-4.99613266530558
H	0.30621752190011	0.65650382250020	-3.36366569969070
C	0.45578721937743	-3.01878237130038	-4.50764878468164
H	0.95528622391383	-3.47308707135040	-2.47136075041714
C	0.11398462506148	-2.03833250731454	-5.42364199893107
H	-0.09465805174965	-2.29362205219530	-6.45342934796097
C	3.19707485559935	-3.47612785223362	-0.32495339826378
H	4.01405843460279	-4.03397947845871	0.15155100728280
H	2.24983819934734	-3.82398090903133	0.09250323629509

H	3.20464703276843	-3.72852644264210	-1.38786021743334
C	4.56419105606095	-1.56125465145280	-0.77585151103754
H	4.62842209499308	-1.90359576701684	-1.81101549899242
H	4.58334651416990	-0.47021089896647	-0.77802399654202
H	5.45799366202571	-1.92585549133035	-0.24702757011669
N	1.00651951242927	-1.10226822758663	-1.43172436078203
H	1.87218690050275	-1.55908710081564	-1.06703535125943
N	1.07403792433127	-0.13005587927581	0.62733807471252
H	0.64880087278101	0.59885195969320	1.17970693403508
N	3.32525622000303	-2.03332474642991	-0.18596405584348
S	-0.70730375878588	0.93781335527012	-0.97037338696295
C	-0.32024084765968	0.36461311908698	-5.96410984151465
C	0.49165426639691	-4.46338054353974	-4.91578687015301
F	-1.62256063607167	0.67404585441560	-5.86580412916533
F	-0.10143359832090	0.00364818173947	-7.23450165672988
F	0.36418509492324	1.49337947215760	-5.74675540156762
F	0.55317298379597	-4.61513966615441	-6.24124780881419
F	-0.60262653350679	-5.12255109630998	-4.49599684421491
F	1.54698403684416	-5.10194163742835	-4.39022225904828
O	-0.74614891483388	-4.43245254186762	-0.45028568846011
O	-1.15907491725108	-3.06457725413785	1.16097393840434
N	-1.33425739544286	-3.47702111668355	0.02522438777194
C	-2.28927823942305	-2.75605494854297	-0.76801313586421
C	-2.65524878768565	-3.21351780747871	-1.96680281777278
C	-3.55326919440456	-2.52423651872703	-2.87508050043226
C	-3.60475850036332	-1.14348781708385	-2.87511388334218
C	-4.42769920517910	-0.44749760287400	-3.76899997204598
C	-5.20071513035609	-1.13252555591805	-4.66608555669702
C	-5.18275384465215	-2.54311867522217	-4.70978328253743
C	-4.34968901123478	-3.26193017878573	-3.80683129104471
C	-4.36848052962280	-4.67282578143738	-3.86283550829680
C	-5.14933980215279	-5.33377337307759	-4.77294193479723
C	-5.96379849921274	-4.62069308485400	-5.66973910785994
C	-5.97948036318035	-3.25447516458975	-5.63388423171590
H	-3.76464189284810	-5.24893736132997	-3.17299387764891
H	-5.14158765269536	-6.41728302466139	-4.79993236640530
H	-6.57846031435415	-5.15646067923683	-6.38361528874207
H	-6.60793260605535	-2.69194421251454	-6.31620627466508
H	-5.83445881653976	-0.59903442814958	-5.36673586649420
H	-4.42936543033472	0.63568615396587	-3.75634094206543
H	-2.95531197651378	-0.58410081471579	-2.20921709611014
H	-2.23182175949854	-4.16489227243513	-2.27165359062314
H	-2.63966701254451	-1.86095476628172	-0.27750880577230

4

C	3.35417262573214	-1.67892752342223	1.06745788681581
C	3.60167021845455	-2.84169456057686	2.02914002126893
C	3.39750572902228	-2.44791014964080	3.48290571255245
C	1.98698859963644	-1.92082548703916	3.68289105374435
C	1.73683987731758	-0.72708789513497	2.77962148078112
C	1.98763398531657	-1.01613356409986	1.29962703810336
H	3.58326973488968	-3.31036693172873	4.12913453188364
H	2.91168943530144	-3.66007864076915	1.80084916832966
H	4.61273777995663	-3.23040806437945	1.87199786019385
H	4.10191835213426	-0.90561312140354	1.27847526918966
H	1.26286074661522	-2.71163270271758	3.45683219840141
H	1.82268787076176	-1.63193594326629	4.72463136280765
H	0.71386328978435	-0.35831200462068	2.89171588666080
H	2.40559731753691	0.08961968846438	3.08600745523257
H	1.20380266028586	-1.68903747147298	0.93293569043804

H	4.12330997799570	-1.67755460417933	3.77158103759029
C	1.26550539627539	0.52545492841772	-0.58237291917721
C	0.61580116460420	-0.72180316781247	-2.63424289836605
C	-0.55514192402528	-0.04863570802016	-2.98763519989873
C	1.11814152043734	-1.68734126703725	-3.50681136435004
C	-1.18437052450591	-0.34414079214491	-4.18524927504683
H	-0.95761668480264	0.70414398261299	-2.32593198482619
C	0.47175203179579	-1.97190097770337	-4.69679573371800
H	2.02529520424024	-2.21970476675623	-3.24909792974270
C	-0.68629830245430	-1.30179842025371	-5.05597904978009
H	-1.18994691554067	-1.52305103955302	-5.98693893015187
C	3.15758948013448	-3.40073945528000	-0.69983570238916
H	2.14055526330285	-3.62414279979064	-0.37430162671489
H	3.18993820469399	-3.51197503480530	-1.78645214605093
H	3.83850567494098	-4.15364564072844	-0.27862692498479
C	4.84392989881839	-1.71082028370958	-0.85963186897308
H	5.05181410852145	-0.65076974801233	-0.70621946733969
H	5.64300381539300	-2.29800942944115	-0.37914732897113
H	4.88716788733663	-1.91082028955993	-1.93284475447905
N	1.28361248550798	-0.53629864783601	-1.43454228323897
H	2.04513650903194	-1.19774449559559	-1.21267697540741
N	1.87022690511138	0.26431508650501	0.60293569410592
H	1.88610506048766	1.08253389926035	1.19192398244734
N	3.52260388475040	-2.03531141441403	-0.35710428257271
S	0.66344215811307	2.04615332516993	-0.89401350668512
C	-2.41789256444057	0.42661462610723	-4.56780562103055
C	1.01021132331048	-3.06446374371027	-5.57378543974137
F	-2.11086768165006	1.54768544101260	-5.23649006699671
F	-3.13459625470973	0.79414348816515	-3.49903534245278
F	-3.22713436774742	-0.28832842244755	-5.36175696311287
F	2.34831389480492	-3.12184060860973	-5.54403198371556
F	0.64381462354409	-2.91900231441055	-6.84983060136709
F	0.56850994158022	-4.27496754735342	-5.17847153299035
O	-0.34914069086386	-3.54513378948638	-0.49729865608937
O	-1.28991295294452	-2.78588963917546	1.28513832085430
N	-1.31141824735626	-3.10878700465505	0.10983676078437
C	-2.56877748655081	-2.94965738663394	-0.56693326534972
C	-2.67209255126567	-3.21662558497447	-1.87036871631319
C	-3.86426961240683	-3.05151188231480	-2.67916501084773
C	-4.86856047663099	-2.19091920645024	-2.27807360907484
C	-6.01640711670881	-1.99148474909810	-3.05064418090586
C	-6.16239841743434	-2.65344164617283	-4.23963596383945
C	-5.16722651567158	-3.53832739500726	-4.70307513555376
C	-3.99538174318986	-3.75120462976289	-3.92185634877808
C	-3.02665202494803	-4.65391487370084	-4.41358215229766
C	-3.19704381919280	-5.29027333770290	-5.61376932229923
C	-4.35191946188953	-5.07181626118806	-6.38489661481034
C	-5.31597420910792	-4.21532629697521	-5.93386813169306
H	-2.13051406757114	-4.85763369885720	-3.84223325736242
H	-2.43171479364283	-5.96898787399034	-5.97175269662078
H	-4.47348249154193	-5.58363497519095	-7.33227242288651
H	-6.21415275029500	-4.03890773304128	-6.51596543380987
H	-7.04495809462004	-2.49764329374583	-4.85089201085965
H	-6.77663414998239	-1.29866341124235	-2.71090197304972
H	-4.74920353522378	-1.62354825579630	-1.36276084315777
H	-1.76869809067652	-3.58171638145978	-2.34588422308923
H	-3.34534511788623	-2.60556247965564	0.09817426077465

5

C	3.01870343020863	-1.05785675023832	1.12941861148524

C	3.90307092414703	-1.89218545571496	2.05831912156554
C	3.61775460855441	-1.64217458750798	3.52890679509362
C	2.16116915246750	-1.95375663123577	3.82286228194413
C	1.25663011876253	-1.09323712278478	2.96176086088481
C	1.52493389471619	-1.20526490739932	1.45930156902328
H	4.27896685687997	-2.25809663849189	4.14464126603284
H	3.73762107441390	-2.95635657076308	1.86224977636296
H	4.95358013834953	-1.69162603591766	1.82566698915296
H	3.26176146745603	-0.00074721108255	1.28437095553448
H	1.96506950229367	-3.01388820248539	3.62594307650769
H	1.92882287008714	-1.78308849870726	4.87749752672602
H	0.20514263026462	-1.33658104884629	3.13678052088869
H	1.39708736783055	-0.04275531627716	3.25130976206353
H	1.18235274732722	-2.19078954611111	1.12117459831041
H	3.83144393251899	-0.59738059481735	3.78570263631407
C	0.04973782074047	-0.18789642406592	-0.35975643202582
C	0.33130385204456	-1.08759852251542	-2.65407547176661
C	-0.35991984650965	-0.15420070898811	-3.42383539492813
C	0.73557575730291	-2.28281843652892	-3.24336492085625
C	-0.64957059293659	-0.43758800547633	-4.74901528119387
H	-0.67587093804942	0.77662726645168	-2.97478324381137
C	0.45169233249380	-2.54183567541161	-4.57435237572201
H	1.25035087068455	-3.02473657166447	-2.64458116285641
C	-0.25143968917133	-1.62679188100266	-5.33989080236239
H	-0.48472549997307	-1.83558264645954	-6.37493216650917
C	3.50117334611382	-2.72520677536191	-0.63612117378892
H	2.70215701521527	-3.35666587562447	-0.24148763508348
H	3.50328267579993	-2.83005897257902	-1.72353794531275
H	4.46215223343760	-3.10788296913532	-0.26748980051037
C	4.30379781826394	-0.47966917772526	-0.86171269590980
H	4.04591430385376	0.56994511451498	-0.71169803599906
H	5.29528660701856	-0.66299382603724	-0.42015175177925
H	4.38060880398077	-0.65960469156337	-1.93623976484727
N	0.69948410153259	-0.87407120775316	-1.32212554440690
H	1.67254094270892	-1.14158211792971	-1.05642036347465
N	0.68572924220155	-0.17026460720837	0.84293476103102
H	0.14425430786543	0.36279588768925	1.50621663336139
N	3.26875152994010	-1.32851017931684	-0.30131976795404
S	-1.41160318042978	0.61583826169256	-0.51445267441759
C	-1.35805713271512	0.60104767855753	-5.57410621183465
C	0.86247031785550	-3.86388039585249	-5.15581185768527
F	-2.26648833892569	1.27449354034590	-4.86140619571858
F	-1.99096742806969	0.06225326191736	-6.62322921666739
F	-0.49662740557824	1.50850407303168	-6.06367810008615
F	0.86262143715748	-3.85274182589180	-6.49126389404808
F	0.03429512453372	-4.85120466797972	-4.77194101914636
F	2.09223212389033	-4.22078927240576	-4.75800781696787
O	-0.14542571239020	-4.54344276726466	-0.59102791931192
O	-0.85887978053326	-3.50893842084179	1.15784419565837
N	-0.94880054541336	-3.82945438418131	-0.01643168932483
C	-2.07551950225380	-3.29904466631160	-0.73147434307554
C	-2.33875584374950	-3.70466868731401	-1.97488327890254
C	-3.39871232915533	-3.17212325671598	-2.81161807309420
C	-3.79550648264098	-1.85682517220276	-2.66432379658292
C	-4.77865828469490	-1.29783152529527	-3.49028374078360
C	-5.36659144708366	-2.05465509549427	-4.46674503482400
C	-4.99672088528215	-3.40296807738842	-4.65981329260092
C	-3.99853389068077	-3.98225834750271	-3.82666023032590
C	-3.66473666752196	-5.33891774283774	-4.03191982495667
C	-4.26756087448375	-6.07376428346819	-5.01752135354613
C	-5.24595383871757	-5.49589785148061	-5.84514480960431
C	-5.60219739804574	-4.18869637409493	-5.66519146761697

H	-2.92708196569883	-5.81543885671082	-3.39851256143529
H	-3.98973356941655	-7.11184328117982	-5.15885873731747
H	-5.71638664363660	-6.08950864194209	-6.62024256341909
H	-6.36002233515509	-3.73203430334088	-6.29296042444237
H	-6.12263611418870	-1.62512318254082	-5.11561420369093
H	-5.05220461557167	-0.25755150075335	-3.36149788220809
H	-3.29750598630306	-1.22661330788740	-1.93434921151771
H	-1.69409150486720	-4.47894338660678	-2.37777648234809
H	-2.63025601107027	-2.57972941798622	-0.14893929934092

6

C	2.03829489431850	-0.74665461421145	1.84012288832494
C	3.48553573480655	-0.93448127284475	2.29983810003779
C	3.60184382890243	-1.77872026951380	3.55696297997321
C	2.98045313236823	-3.14206053120656	3.31149808432641
C	1.52367070688621	-2.99122508408460	2.91741439495816
C	1.28993873334549	-2.08263506175600	1.70842091014414
H	4.65302094328558	-1.87624040043851	3.84190690817480
H	4.05867358921027	-1.43071761584675	1.51020081762113
H	3.94335012347512	0.04870777219719	2.44705612181785
H	1.50696966299807	-0.17502163515134	2.60922244417913
H	3.53009960620008	-3.65882640527983	2.51654230304327
H	3.05255017194971	-3.77188784168930	4.20228135916026
H	1.07565005851513	-3.96484493580004	2.70102021357543
H	0.97518898538691	-2.56611228785240	3.76921661996562
H	1.64796589162871	-2.60368725854330	0.81211956985842
H	3.09315548233490	-1.28603098673271	4.39464325625875
C	-0.94920362759313	-1.79961712221273	0.51498931195076
C	-0.87770380308195	-0.83671838216764	-1.77007109006807
C	-2.21881145469139	-0.54304273998306	-2.00817232367725
C	0.02601713731781	-0.77407354163455	-2.82774685872843
C	-2.63210091460177	-0.21536487413533	-3.28959053564618
H	-2.92632445004966	-0.58735072836679	-1.19249285911777
C	-0.40250659758419	-0.42843807092393	-4.09901103830278
H	1.06567706248560	-1.02631129176782	-2.65613031362163
C	-1.73712202312494	-0.15393288247884	-4.34651068248422
H	-2.07321769727429	0.10252919084086	-5.34183465719724
C	1.81310648509817	1.46724843846677	0.84086655706085
H	2.71666695377355	1.89694043764803	1.29979123028574
H	1.64145855315411	1.98724573365900	-0.10414408449892
H	0.96331569803083	1.66591067594560	1.49591450152606
C	2.94254926597230	-0.23637964791466	-0.40757510709964
H	3.94231543628641	0.11966819908524	-0.12554835639347
H	3.00165836948122	-1.30729245565700	-0.61368200615402
H	2.66628668335054	0.26759351171997	-1.33663763869737
N	-0.36322526021496	-1.14112106886447	-0.50591107268416
H	0.53176129922610	-0.67526804252267	-0.23986697933981
N	-0.16736385398132	-1.92879543521697	1.62100556286985
H	-0.67296660982237	-2.42460690788260	2.33920823651212
N	1.92605837504163	0.04144139097478	0.59553335964011
S	-2.49624616143275	-2.44105491009172	0.53826275606448
C	-4.07497367064857	0.14190003407558	-3.51752117903511
C	0.59531285065593	-0.42154095962418	-5.22111699191773
F	-4.44380939440791	-0.04888326575359	-4.78999591044370
F	-4.30963096247932	1.43424044851890	-3.23438472165647
F	-4.89841234996724	-0.57853913103966	-2.74982951447627
F	0.86624669312766	-1.66704031421143	-5.64912643098695
F	1.76425881876900	0.11544932799562	-4.84337866676565
F	0.16276534890726	0.26840027437104	-6.27981332120659
O	1.88624008585155	-3.78095433707910	-2.19957343202175

O	1.28605057852318	-4.70352972227378	-0.34850718220741
N	1.06363568259125	-4.33336880410382	-1.49025770731204
C	-0.26451168988292	-4.56859787851493	-1.98297906426199
C	-0.56416471572481	-4.32550912504761	-3.26015052575705
C	-1.89028693315654	-4.44894263592506	-3.83725810698252
C	-3.00510667212912	-4.22606964805551	-3.05193130653025
C	-4.29660097733547	-4.28586640676251	-3.58992581839312
C	-4.47510387650072	-4.56831752689207	-4.91669572074479
C	-3.37003932652218	-4.80281353473143	-5.76277051204124
C	-2.05241589099998	-4.74471123959051	-5.22749593666438
C	-0.96900570212910	-5.00334148826019	-6.09550809685243
C	-1.17554473601107	-5.28208423343235	-7.42008854147287
C	-2.47763056107150	-5.32896772573458	-7.94778590978117
C	-3.54969704615288	-5.09640514388853	-7.13254801934185
H	0.04408120353571	-4.99120181976256	-5.71357490353048
H	-0.32683899303103	-5.47133102597710	-8.06714126714601
H	-2.62681430381085	-5.55251111893532	-8.99765154649814
H	-4.56018871466686	-5.13530318283778	-7.52537035310461
H	-5.47254879023918	-4.60752022891589	-5.34177436118388
H	-5.14823601923871	-4.08601407426743	-2.95087871289567
H	-2.87831813080754	-3.94277564726577	-2.01192212964147
H	0.25317989274354	-3.99835391783876	-3.89439285768089
H	-0.92655210916889	-4.94298999600459	-1.21744513508352

7

C	1.66317126699373	-2.02466243086576	1.66153718966507
C	1.67738354231121	-1.35004403858628	3.03629968786989
C	2.95364681918442	-0.54720843874313	3.25257966408001
C	3.15058801818183	0.47835874125135	2.14629314021183
C	3.10759488508665	-0.17801493969657	0.77513604792806
C	1.80709083104369	-0.95407546265461	0.57591143366509
H	2.92757278725726	-0.05823725986606	4.23077829968181
H	0.81197247816850	-0.68318358253073	3.12236579823417
H	1.57191932345844	-2.10723307101577	3.81842214898127
H	2.55700696787021	-2.66250779004650	1.59922469077066
H	2.36173935982597	1.23781698179145	2.20742477325963
H	4.09900802638250	1.00662088799500	2.27729330504862
H	3.19346949696441	0.56888180603791	-0.01650397597194
H	3.94920594206822	-0.87223077999044	0.66355023493690
H	0.97759200840360	-0.23995919570127	0.64164804569677
H	3.81159059406179	-1.23076541647489	3.27002453448128
C	1.38550727253724	-0.95499008855554	-1.86158626431101
C	1.10771387103392	-1.63159064783821	-4.28930767945533
C	0.87784757720637	-0.41150478228749	-4.91809594554995
C	1.09559162455215	-2.79894808111256	-5.06233196018446
C	0.63407456373427	-0.38117000257934	-6.28473616071797
H	0.89505794102446	0.49868771473931	-4.33612324796700
C	0.85320365074030	-2.74370833000342	-6.41936902537742
H	1.28970680228107	-3.75654548404282	-4.59112142352245
C	0.61349777968999	-1.53081040837289	-7.05145970304126
H	0.42832235381276	-1.48695513640993	-8.11575218051888
C	-0.76821352831276	-2.30205129455101	1.48809423787138
H	-1.05510272075343	-2.02256900753115	2.51475666806988
H	-0.81808426057769	-1.40770577315101	0.86477477837861
H	-1.51970088695379	-3.00404171882355	1.11846837274378
C	0.59859005164857	-4.14360604481439	2.16152873198967
H	-0.16814408629410	-4.83430654389140	1.80154962164121
H	1.57047417319037	-4.62291871144282	2.02471980186413
H	0.43116137849886	-4.00422970620126	3.24257317748257
N	1.37460523766558	-1.80978775300837	-2.93507794361509

H	1.59746502611887	-2.76946973274185	-2.70454788601207
N	1.74637972036339	-1.58215996797992	-0.72637214863343
H	1.73150147091032	-2.59512475447443	-0.71218523776634
N	0.53837874600220	-2.91719497075800	1.39891206311017
S	0.98046293588850	0.66227609689482	-1.90174330968988
C	0.37025058444605	0.95820684290801	-6.91748449002157
C	0.79554062149536	-4.01532348943012	-7.21768682475844
F	-0.79804330274540	1.47263827937953	-6.50386281716734
F	0.31707255531607	0.88645069560509	-8.25176998600639
F	1.31821619760390	1.84960781764323	-6.60387411959772
F	-0.46653886998207	-4.45902776036304	-7.34112668809113
F	1.49882502984141	-5.00172566882203	-6.64917071784306
F	1.26957144109255	-3.84773313310287	-8.45695450069921
O	1.34615327503553	-4.59242999569886	-1.75761143825975
O	1.05243108784226	-6.43817211038437	-0.69630335961448
N	0.62770766061848	-5.46976418869849	-1.28916244844014
C	-0.79589988948804	-5.37953403066580	-1.42501084953814
C	-1.36956178523573	-4.30684134595460	-1.97780586377325
C	-2.78972310064645	-4.11140621579029	-2.18379578295263
C	-3.64702231128942	-5.19596021603735	-2.22918535469915
C	-5.01610932083414	-5.03825089007231	-2.45944855189649
C	-5.53529447777201	-3.78536806805868	-2.65238087560439
C	-4.70931553518732	-2.64345600855575	-2.62041376258828
C	-3.31265910456526	-2.79176075825309	-2.38011658208852
C	-2.52172736883487	-1.62314904869280	-2.33235976451436
C	-3.07191680472985	-0.38595256966163	-2.53497964510452
C	-4.44827643060378	-0.24583547657909	-2.78439199561261
C	-5.24799241324297	-1.35299980564339	-2.82148286190558
H	-1.46113074616360	-1.68034318930181	-2.12531088492342
H	-2.43114116032648	0.48752647592954	-2.49977110498393
H	-4.86976489651852	0.73961124069439	-2.94369318991274
H	-6.31282785291236	-1.25989267447872	-3.00627309490332
H	-6.59565289789916	-3.65411410465180	-2.84063316007866
H	-5.65740745322946	-5.91036826641147	-2.49957610885108
H	-3.24598187697824	-6.19701503570654	-2.12171106352355
H	-0.69900378649568	-3.51544594230725	-2.29039998320425
H	-1.29183910888063	-6.24002124080390	-1.00348348416976

8

C	1.35361745468510	-1.18982807613901	1.74127515801044
C	1.70247409509119	-1.36502864837074	3.22358720157627
C	2.65262293489393	-2.53570370989385	3.44018985839517
C	3.92284043541823	-2.36886830876575	2.61999427834074
C	3.60024574323265	-2.16344766796259	1.14766058237349
C	2.64814093274094	-0.98590759385895	0.94950578718718
H	2.89269822947000	-2.63251241673623	4.50327426982666
H	2.16804381304616	-0.44656139233273	3.59970614687917
H	0.78629815205567	-1.51430933692978	3.80174458447443
H	0.91093141740709	-2.13414117642112	1.39528572482760
H	4.48482540678493	-1.50298993189518	2.99063843525661
H	4.57551976995603	-3.23812390994153	2.74128571898035
H	4.50981440867184	-1.96992951314978	0.57335203827830
H	3.13512280926384	-3.06748847654506	0.73755064615484
H	3.15810839060425	-0.08067973962377	1.29103090702837
H	2.15142999913314	-3.46597628125716	3.14631705080742
C	2.99181079776190	0.10683801356422	-1.22185193651551
C	1.23609808248926	0.09227923962709	-3.02458066234077
C	0.08074781093942	0.06701073982801	-2.24154854678709
C	1.10917515919762	0.01596579298993	-4.41347294040809
C	-1.15773347307817	-0.05012630136485	-2.85454063597582

H	0.14114921372290	0.15049658751305	-1.16027945310806
C	-0.13636480299524	-0.08672005972548	-5.00250442953770
H	1.99951888881301	0.04071673894107	-5.03160355734230
C	-1.28835025481921	-0.13124515401413	-4.23115816814451
H	-2.26247148485048	-0.20911123496037	-4.69294623877058
C	0.73145926112896	1.18836530945751	1.80810782609923
H	-0.01876961401140	1.87968584215687	1.41626523340525
H	0.78942568901633	1.34924979371292	2.89733314752955
H	1.69201717045205	1.46326418210992	1.36897172047893
C	-0.95806182856879	-0.49568424943780	1.92340683900919
H	-1.05308875408602	-0.45443310213485	3.02170293529689
H	-1.68722115003692	0.20163397698374	1.50515353451692
H	-1.23060591074887	-1.50230619572056	1.59750969327923
N	2.51725893618885	0.25187319765524	-2.50982784266509
H	3.17043066253377	0.75787914418712	-3.08696045321040
N	2.35481099585104	-0.77163756752811	-0.45096809722474
H	1.67400402497150	-1.39398981196652	-0.86973332871130
N	0.36174854059408	-0.16125811441195	1.43917548793091
S	4.32570750757519	0.99985494909174	-0.77052547131509
C	-2.38191324940221	-0.10055860230902	-1.98232984176981
C	-0.22422003331215	-0.21123891897535	-6.49663671851077
F	-2.37275151435405	0.86354576806903	-1.05354209536597
F	-2.46919940314704	-1.27059505745692	-1.32627222119226
F	-3.51092803289301	0.03561656142844	-2.68465164015058
F	0.00508581659805	-1.47914334128871	-6.89501486654571
F	0.67967215485405	0.55223104418347	-7.11920211377775
F	-1.42862243250178	0.12688159993463	-6.96418777374474
O	0.49947352723819	-3.06005355997958	-1.67999556844375
O	0.61369804635267	-4.42807302918460	-0.01910018030016
N	0.16544449238208	-4.07813432871012	-1.09529415767010
C	-0.81656970950445	-4.94403673107251	-1.68429819332235
C	-1.41533465415397	-4.61155486223430	-2.82966502189158
C	-2.45638121529660	-5.37439409038436	-3.49131661181080
C	-3.23006009362404	-6.26839228433971	-2.77498323537938
C	-4.25803103730458	-6.99825275776933	-3.37915733108215
C	-4.52212608711143	-6.82989121144085	-4.71185411955476
C	-3.76932942080648	-5.92786066423296	-5.49146516875216
C	-2.71601411612482	-5.18317589037257	-4.88660899748111
C	-1.97062147256970	-4.30562663542783	-5.70493883169120
C	-2.26362786246591	-4.15719174839036	-7.03420455013484
C	-3.31084369157096	-4.88639297257312	-7.62407084602105
C	-4.04394454286344	-5.75496421115965	-6.86632154912464
H	-1.14447466727950	-3.74134775666040	-5.29169665998046
H	-1.67863263151624	-3.47121607000220	-7.63506808612614
H	-3.52972804874285	-4.75936799559071	-8.67769982374616
H	-4.85057561849835	-6.32940379314040	-7.30909812608990
H	-5.32254383999448	-7.38604210403546	-5.18821541768031
H	-4.84986954542012	-7.68353264832533	-2.78443502385114
H	-3.06296542034983	-6.38287996407437	-1.71048424694189
H	-1.08255335216053	-3.68401673491202	-3.28178870904677
H	-0.97516180495232	-5.83276254630335	-1.09361231670569

9

C	1.86699332702848	-1.99834010529390	1.75078155285799
C	1.80818995314305	-1.35180159480476	3.13702509804382
C	2.95992049539619	-0.37702177327417	3.34635662133643
C	2.98939185707489	0.68042057803213	2.25283182317040
C	3.01541312523828	0.04426946136605	0.87154383144696
C	1.83241255021079	-0.90263244153466	0.68069690933654
H	2.88101356723271	0.09057522706935	4.33213541956695

H	0.85779488840027	-0.81781127117251	3.24928332416297
H	1.82680562693772	-2.12923128469838	3.90619140810914
H	2.84424020639314	-2.49422412894865	1.66082684681513
H	2.10234691586327	1.31967746971965	2.33845985043199
H	3.85559191420361	1.33612488017171	2.37711589810638
H	2.98430427631898	0.80713977911749	0.09166378113158
H	3.94274385565997	-0.52541126290213	0.73662133507655
H	0.91144563462116	-0.31375210986117	0.77372044309128
H	3.90689147284624	-0.93089929073933	3.34031608333984
C	1.40475294294151	-0.93302984110260	-1.75778198317820
C	1.18069891509017	-1.62117960843676	-4.18511824682970
C	0.85399343764282	-0.41951205070739	-4.80765902550059
C	1.24668932042770	-2.78414146765701	-4.96208211814358
C	0.58699133697963	-0.40468385072203	-6.16966356997140
H	0.80850792115546	0.48754740701415	-4.22259387045762
C	0.97833919015305	-2.74421084978635	-6.31532693941741
H	1.50956311004185	-3.72730608234101	-4.49518532463981
C	0.63997514449719	-1.55158200267390	-6.93980813908921
H	0.42370418474826	-1.52165956807005	-7.99875122578529
C	-0.49611537273054	-2.64151494321410	1.64981667566999
H	-1.14686733712549	-3.43445476401607	1.27356555363285
H	-0.77946691712289	-2.45217208889806	2.69767796590563
H	-0.70497976655035	-1.73895212925106	1.07248088095940
C	1.16198840096651	-4.27495016408350	2.19660543462485
H	1.01074511852334	-4.20067636002450	3.28634559836090
H	0.50884441624989	-5.06652508175666	1.82323880155837
H	2.19348732544570	-4.58404111960788	2.01470764745576
N	1.47123497972122	-1.78103158942734	-2.83377243720123
H	1.75067298231328	-2.72773637986325	-2.60219328533632
N	1.83614450535813	-1.52417450342493	-0.62682340393579
H	1.89335579628465	-2.53594161822166	-0.60584053116848
N	0.88396359535084	-3.04477203317736	1.48809188999286
S	0.84286428392745	0.63719833571411	-1.78683345193627
C	0.19281684734440	0.90662909275156	-6.79287350653396
C	1.10091992679692	-3.99788817377105	-7.13482436954358
F	1.00556455888197	1.90035973152410	-6.41704159052082
F	-1.05044620740986	1.26447291463720	-6.43183965384417
F	0.21261660111099	0.85809921230646	-8.12913383571723
F	0.20806752022114	-4.02989895408154	-8.13083045991444
F	0.91842567841513	-5.10117926655831	-6.39879423975746
F	2.31508852916720	-4.10089606351598	-7.69702077836262
O	0.25158733049909	-6.05963344790815	-0.72112149153215
O	1.46135639998642	-4.62731826566664	-1.77454396471652
N	0.37305974044464	-5.08284122751260	-1.42462267690678
C	-0.78434763618162	-4.38249824818236	-1.90217603622373
C	-2.00896340205179	-4.83769998689606	-1.61581376977437
C	-3.29293408161507	-4.32273061089350	-2.04040551952750
C	-4.31694702063677	-5.25490581749903	-2.07524092194421
C	-5.57486972628044	-4.95452277634555	-2.60702793976313
C	-5.81736537566005	-3.70024358537731	-3.09582965619984
C	-4.83357433198020	-2.68987396164404	-3.02886867428229
C	-3.55438872979970	-2.97950487725931	-2.47101084773732
C	-2.64304793059298	-1.91155513311059	-2.33372211508650
C	-2.94651724336016	-0.65148015395013	-2.78023518693207
C	-4.18528381974123	-0.38726749252267	-3.38577394272666
C	-5.11317784596061	-1.38571497261360	-3.49179327854814
H	-1.69789495144095	-2.06295091620163	-1.83274165703277
H	-2.21777628296716	0.14139001840842	-2.65411179294013
H	-4.40815543484166	0.60947935311131	-3.74740193937696
H	-6.08764776629251	-1.18994813243042	-3.92636687092323
H	-6.78140386962357	-3.45580352596207	-3.52934093166461
H	-6.34203189504219	-5.71839682240825	-2.64057418801062

H	-4.11920543176149	-6.25891819823940	-1.71546809137623
H	-2.03711052240487	-5.76056850006024	-1.04228755682885
H	-0.52922380808115	-3.53848899063946	-2.51951363734421

10

C	1.76002568545049	-0.91664590226478	1.42145285597057
C	2.00394408988890	-0.71624735710064	2.92143363327653
C	2.36340114430407	-2.02661557532165	3.60967268698704
C	3.58059598014094	-2.66952302260060	2.96228173635056
C	3.37434183020302	-2.84934988359373	1.46601432951927
C	3.01580620187659	-1.52625817976374	0.79282198854977
H	2.54224948201665	-1.85478368112330	4.67531323007186
H	2.81775241534137	0.00375147201748	3.06689640630509
H	1.11258591071251	-0.28306633001184	3.38363812381725
H	0.95439997816828	-1.65590598961221	1.31505135169013
H	4.46001916237254	-2.03620099227478	3.13063151050729
H	3.79942561210832	-3.63477205979780	3.42787889266595
H	4.27734232276782	-3.24284459959952	0.99243204005150
H	2.56708743148542	-3.56912554140409	1.28374420348341
H	3.86511517547474	-0.84769290418033	0.91303914708268
H	1.51136050813305	-2.71421773714096	3.54303944095664
C	3.78805509307875	-1.47651902124330	-1.53782814651911
C	2.18558312824615	-1.37288023051603	-3.47399399177571
C	1.15251973374725	-0.63335879363095	-2.89661554285468
C	2.01300821700506	-1.87627762150689	-4.76597535510806
C	-0.01950316180569	-0.42486299909211	-3.60929644659129
H	1.26106680352451	-0.21160911708428	-1.90155567536234
C	0.84075811249623	-1.64766208667329	-5.45983518485774
H	2.81007437228396	-2.45002577142431	-5.22460321832713
C	-0.19620121594292	-0.92381957885440	-4.88862489318901
H	-1.11175483929665	-0.74229231019039	-5.43399459001586
C	2.20675295125025	1.38968062148756	0.69404223967260
H	2.31943442343242	1.86313476839741	1.68345178015437
H	3.19653535228338	1.09674977917590	0.33940899316617
H	1.82502796223450	2.15192198311289	0.01014587915372
C	-0.03883089228631	0.66000357302010	1.05826044722866
H	-0.11018157860454	1.09527171445992	2.06945228298709
H	-0.40217227024067	1.41085991391282	0.35302063223704
H	-0.70889347772454	-0.20142754144632	1.01074965278393
N	3.41327315313273	-1.59513996717386	-2.86119608973932
H	4.21582730295182	-1.63838214257599	-3.46923861293868
N	2.83577265909695	-1.69079653247642	-0.63307159099871
H	1.94881614711589	-2.07725356499789	-0.93321949884515
N	1.30104175936133	0.26130148965514	0.68990263752697
S	5.38209366723509	-1.12486143537096	-1.19881337014191
C	-1.12329569148420	0.35549517721078	-2.95072009724389
C	0.66190665866259	-2.25241061893586	-6.82323907661758
F	-2.08868013225087	0.69463138754288	-3.81010037701934
F	-0.67271109947495	1.48469526448594	-2.39163634761476
F	-1.70780218876527	-0.35598977514015	-1.96927803512931
F	0.13210409306288	-3.48912457477620	-6.74094595773134
F	1.82079067284186	-2.37502320913012	-7.47809483623519
F	-0.16085677642366	-1.53206738415360	-7.59017341528943
O	-0.97491369112352	-3.24490183773312	0.34401122428000
O	0.26092332214511	-3.34488998883665	-1.41746711135768
N	-0.81015209144948	-3.45831799262395	-0.84242947981602
C	-1.97471432669750	-3.86421180915583	-1.58056447492529
C	-1.87853590039828	-4.12803998763132	-2.88655968805553
C	-2.89952588055148	-4.59609550400420	-3.80272872490420
C	-2.43272461114406	-5.32092158974077	-4.88523122243960

C	-3.29991288652226	-5.96332097532148	-5.77612169086800
C	-4.65101232883454	-5.86260543822554	-5.59452282494430
C	-5.18597396240770	-5.07793732957456	-4.54923662850327
C	-4.31172264543721	-4.40803259077896	-3.64537562683048
C	-4.89007184059585	-3.55003630989822	-2.68559786102165
C	-6.24964261375240	-3.41022654485459	-2.58502894838416
C	-7.10909767933988	-4.12093952649474	-3.43809057207883
C	-6.58260952391720	-4.92763796161754	-4.40788592729312
H	-4.25477499950057	-2.95322534667248	-2.04671950978803
H	-6.66417445898214	-2.73334689757757	-1.84698666191919
H	-8.18250277563778	-4.00906647266458	-3.34122716534397
H	-7.23167944638727	-5.45631491620097	-5.09773817008208
H	-5.33668456839152	-6.36713323089577	-6.26699918273136
H	-2.89432517468808	-6.54135810269930	-6.59741234427557
H	-1.36187996236398	-5.41804767461831	-5.02528418244553
H	-0.87797623593867	-4.04288439250297	-3.30208076032849
H	-2.83954458727174	-3.94768969197159	-0.94412823799379

38

C	1.95854869350094	-1.38903739716646	2.31139961950428
C	2.65643155334117	-1.07090445111539	3.63649180485209
C	3.73889006488050	-0.01411970507286	3.45528329118977
C	3.17572997807171	1.24884134032765	2.81969927245289
C	2.45215751278019	0.93660300504667	1.51851693617201
C	1.35620797878086	-0.10290746766111	1.73870746230451
H	4.20504074190814	0.21515454200385	4.41819041148379
H	1.91555535050705	-0.71107450625811	4.35950339059472
H	3.08802888875743	-1.98517172420207	4.05446292371046
H	2.73180089859702	-1.72017313134855	1.60276621418957
H	2.47441763418310	1.72558726339778	3.51512505875038
H	3.97349761037093	1.97489719419774	2.63937669967673
H	1.99946310334282	1.83737233112977	1.09919281299843
H	3.16044241840056	0.54853237718242	0.77644963527881
H	0.63440287987021	0.32126580992157	2.44754716078142
H	4.53277260218980	-0.41831297554145	2.81461899352460
C	-0.38212541404096	0.30892278472685	0.03625283475578
C	-2.00881265227483	0.11140836793716	-1.86339482650292
C	-3.14294633040607	0.73077823150222	-1.33448597867548
C	-2.00917317091783	-0.24798873804553	-3.21039422718081
C	-4.23040196819492	0.98262595438647	-2.15297452191228
H	-3.16252233883786	1.00962402360148	-0.29185158210926
C	-3.11344297545742	0.00083816626095	-4.00811654964456
H	-1.13420389053912	-0.72667863682628	-3.63400271284486
C	-4.23595793345478	0.62549781509943	-3.49408838682851
H	-5.09667128263394	0.82349261178655	-4.11763887648498
C	-0.12689351784694	-2.28357826609345	3.24898021278943
H	-0.87334949151326	-3.05966240643733	3.06305466495153
H	0.15681305677255	-2.34516297423932	4.31266052795316
H	-0.60682753635236	-1.31851552273956	3.07854548800092
C	1.57589682535386	-3.77773134207959	2.48007333212887
H	0.81386529480493	-4.54189268795625	2.30668317147353
H	2.36003329334669	-3.91122758306386	1.73140483232561
H	2.01229069778299	-3.96718927690483	3.47590013769987
N	-0.89114797256949	-0.23762184200563	-1.11667507481179
H	-0.29546658979652	-0.92353381661826	-1.56774566732217
N	0.64223844159591	-0.41766203076330	0.52136510391271
H	0.71730930924618	-1.38574111390764	0.23037212868244
N	0.98229335370294	-2.47031039361418	2.34108559886715
S	-0.91835467823619	1.71681102492680	0.74340815705679
C	-5.42683182140596	1.69305817598310	-1.58246980324479

C	-3.07359899845922	-0.45237375778492	-5.43719158455465
F	-5.57419183566727	1.46598093117535	-0.27400431851559
F	-6.56350574979906	1.31243217195130	-2.18344404728374
F	-5.33203211956438	3.02188426361748	-1.74252481887248
F	-1.93525465041003	-0.09373407722334	-6.04770477029794
F	-4.08463934032308	0.03241410358041	-6.16013192743833
F	-3.13931035619373	-1.79886443341878	-5.52984056304839
O	0.53904590619681	-2.37137499052670	-2.75063423480362
O	1.44526878482303	-3.45404773309781	-1.12681311494868
N	1.03281421129735	-3.38222972824209	-2.26936459350302
C	1.13301665956484	-4.56677460918937	-3.06761225566382
C	0.63894052738712	-4.58108912094021	-4.30927015273013
C	0.67947209749349	-5.69381483335636	-5.23370518792519
C	1.62480645073644	-6.69284270996424	-5.08951083333737
C	1.70558892011532	-7.75946428058164	-5.98881506356270
C	0.83659574393260	-7.82798724960069	-7.04535679097487
C	-0.14737655962709	-6.83760064625918	-7.24269670440097
C	-0.24208173391464	-5.74854645123539	-6.32932897233314
C	-1.25726853254181	-4.79026487929302	-6.54253491187852
C	-2.11070803423997	-4.89066312183616	-7.60844778638204
C	-2.00527627396278	-5.95968537808301	-8.51518004859158
C	-1.04463672101243	-6.91350326847678	-8.33115175577490
H	-1.38739410489622	-3.96353778991142	-5.85638554387274
H	-2.87756422355757	-4.13776308415808	-7.74813897235047
H	-2.68917530774347	-6.02584078698896	-9.35303122829940
H	-0.95665660340740	-7.74873168337017	-9.01761472843098
H	0.89660303788335	-8.64847174548795	-7.75257868005653
H	2.46496814267215	-8.51987453209740	-5.85261620789001
H	2.34559030458897	-6.63133683317483	-4.28249532333281
H	0.16502465519837	-3.66172209639019	-4.63355929095777
H	1.59571608582034	-5.38040667939185	-2.53128325848642

9. Computed IR spectra of the bare catalyst with PBE0

Here we show the agreement between experiment and PBE0 calculations of the bare catalyst based on the assigned **sa1** conformer. Although all features of the experiment are well reproduced by the calculations, the peak positions deviate more from the experimentally observed positions than found for calculations with M06-2X. As a result, also in calculations of the **Cat··Nitro** complex similar deviations are observed.

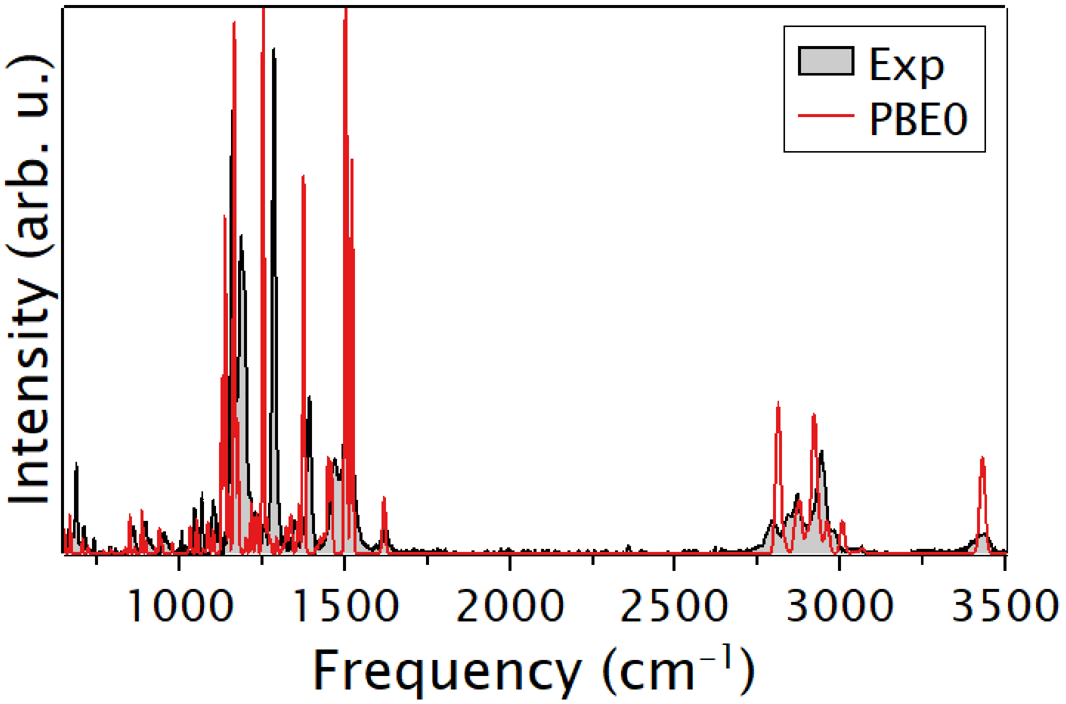

Figure S7. Comparison of the measured infrared spectrum of the bare catalyst (black) with calculations of the infrared absorption spectrum computed with PBE0 (red) for the **sa1** conformer. The same vibrational frequency scaling factor as for the **Cat··Nitro** complex is employed.

10. Comparison of experiment and computations for the 5 lowest-energy conformers

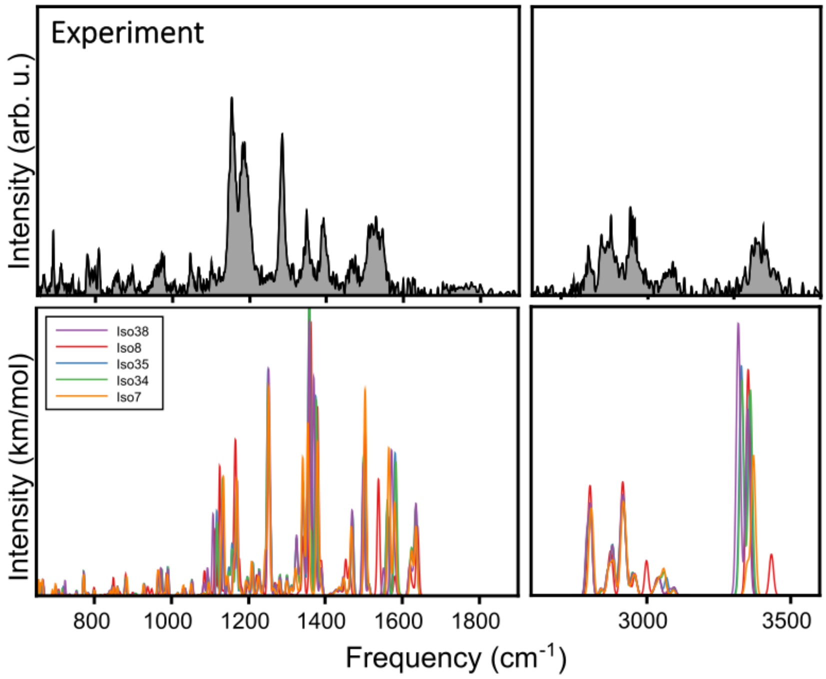

Figure S8. Comparison of the measured infrared spectrum of the complex and the 5 lowest energy conformers at the PBE0 level of theory.

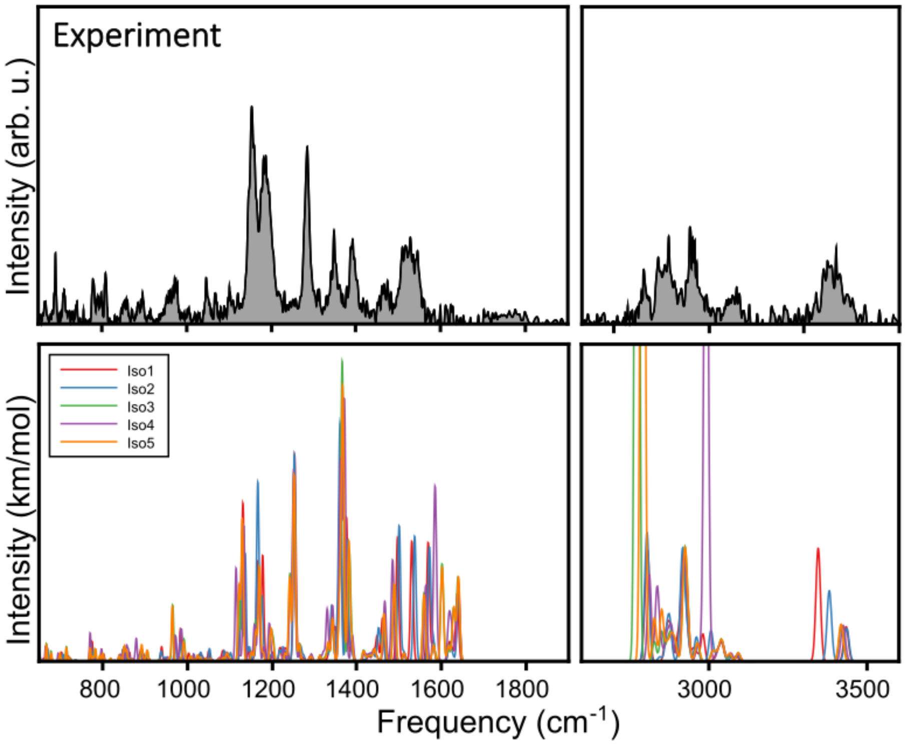

Figure S9. Comparison of the measured infrared spectrum of the complex and the 5 lowest energy conformers at the M06-2X level of theory.
